# Interpretable AI-enabled decision support for drinking-straw substitution using per-use greenhouse-gas indicators and user-review evidence

**DOI:** 10.1038/s41598-026-63847-8

**Published:** 2026-07-29

**Authors:** Marwa S. Hassan, Shymaa Khamis, Ahmed Barakat, Randa M. Osman, Gassan Hodaifa, Jie Tang, Shaoshan Liu

**Affiliations:** 1 Systems and Information Department, Engineering Research Institute, and New and Renewable Energy, National Research Centre, Giza, Egypt; 2 Environmental Licensing Department, Industrial Development Authority, Cairo, Egypt; 3 Basic Science Department, Faculty of Engineering, The British University in Egypt, Cairo, Egypt; 4 Chemical Engineering and Pilot Plant Department, Engineering Research Institute, New and Renewable Energy, National Research Centre, Giza, Egypt; 5 Molecular Biology and Biochemical Engineering Department, Chemical Engineering Area, Universidad Pablo de Olavide, ES-14089 Dos Hermanas, Spain; 6 South China University of Technology, Guangzhou, China; 7 Shenzhen Institute of Artificial Intelligence and Robotics for Society (AIRS), Shenzhen, China

**Keywords:** Drinking-straw substitution, Per-use greenhouse-gas indicators, Review-derived user evidence, Multi-criteria decision analysis, Natural language processing, Interpretable AI-enabled decision support., Climate sciences, Engineering, Environmental sciences

## Abstract

Plastic drinking straws are a visible single-use plastic product, yet selecting suitable substitutes remains challenging because literature-derived climate evidence and reported user experience are rarely evaluated together. This study develops and demonstrates an interpretable AI-enabled decision-support workflow that integrates literature-derived per-use greenhouse gas (GHG) indicators with review-derived user evidence extracted from online customer reviews using natural language processing (NLP). Drinking-straw alternatives were used as an information-rich case study. The integrated assessment combined a GHG-derived score, a user-experience feature score, and rating-based consumer approval within a transparent multi-criteria decision analysis (MCDA) under four predefined decision-priority scenarios. Among the five shortlisted materials and within the evaluated dataset, the selected per-use GHG assumptions, review-derived user evidence, normalization procedure, and scenario-specific weights resulted in Silicone achieving the highest integrated MCDA score across all four scenarios, whereas Paper ranked lowest. Silicone combined a low per-use GHG indicator with the highest user-experience feature score and high consumer approval. Paper had the highest per-use GHG indicator and a moderate user-experience feature score, while lexical analysis identified recurring functionality-related expressions in its reviews. A shallow decision tree identified a 0.081 kg CO₂e/use threshold separating Paper from the lower-per-use-GHG reusable alternatives within the evaluated decision matrix. The study is not a new process-based life-cycle assessment or comprehensive sustainability assessment. Instead, it demonstrates decision support limited to per-use GHG indicators and review-derived user evidence; broader sustainability dimensions were outside the scope. Future studies may adapt and evaluate the workflow for other product categories using product-appropriate environmental criteria and relevant user-derived evidence.

**Supplementary Information:**

The online version contains supplementary material available at 10.1038/s41598-026-63847-8.

## Introduction

Single-use plastics continue to accumulate in natural ecosystems, raising persistent concerns about environmental degradation and potential risks to human health^1^. Among these items, drinking straws are a highly visible category of disposable plastics that are widely used yet difficult to recycle because of their small size and material characteristics, leading to frequent disposal in landfills or leakage into the environment^2,3^. In the United States alone, Reuters^4^, citing Technomic data, reported approximately 63 billion drinking straws per year, equivalent to about 170–175 million straws per day. Previous studies on drinking-straw alternatives have also highlighted the need to evaluate substitution options beyond simple material replacement, including product performance and use-related considerations^5^. Globally, plastic waste generation and mismanagement continue to increase, underscoring the urgency of improving evidence-based decisions about single-use plastic substitution^6–8^.

Several life-cycle assessment (LCA) studies have compared alternatives to conventional drinking straws by examining greenhouse-gas emissions and other LCA-reported dimensions such as resource use and end-of-life assumptions^9,10^. Comparative studies from Brazil and the United States have further shown that conclusions are sensitive to reuse assumptions, system boundaries, and end-of-life scenarios^11,12^. Overall, this evidence indicates that no straw material performs best in every situation. This context-dependence makes the selection of straw alternatives more complex than a simple replacement of plastic with any single material.

Alongside this environmental work, other studies have examined how straw alternatives perform in actual use, including durability, comfort, sensory acceptability, and usability^13,14^. Recent work has also begun to connect material properties, end-of-life behaviour, and consumer experience for straw alternatives. Liang et al.^15^ compared paper, polylactic acid (PLA), and polypropylene (PP) straws using material characterization, soil-burial biodegradation tests, and consumer testing. Their results showed that paper straws exhibited substantial water absorption during use, which could affect drink taste and user experience, whereas PLA straws maintained stronger use performance but showed limited degradation under natural soil conditions. Their consumer testing further indicated that ease of inserting the straw into a beverage and straw fracture were important factors influencing purchase willingness. These findings highlight the importance of evaluating straw alternatives not only by environmental or material claims, but also by real-use performance and consumer response.

The present study builds on this direction by integrating literature-derived per-use GHG indicators with large-scale review-derived user evidence across multiple straw materials. Rather than conducting controlled material testing, a representative consumer survey, or a new process-based LCA, the study demonstrates an interpretable decision-support workflow that combines literature-derived per-use GHG evidence with user-derived evidence extracted from online customer reviews. In this context, Amazon reviews are used as a proxy for user experience, not as a substitute for controlled user testing or representative consumer research.

This practical dimension is important because an alternative may appear favourable under specific LCA assumptions but may be less suitable in practice when usability is poor. For example, experimental evidence shows that paper straws can lose around 70–90% of their compressive strength after 30 min of immersion, raising clear concerns about functionality under use conditions^16^. More broadly, recent consumer research also shows that environmental indicators influence purchasing decisions, with 56.9% of respondents in one large survey considering such indicators important when purchasing lifestyle essentials^17^. The main material characteristics are summarized in Supplementary Table S1, while Supplementary Tables S2a–S2b compare LCA evidence across different regions, system boundaries, and harmonization assumptions.

Despite the expanding literature on alternative drinking straws, prior studies remain divided into two main approaches. As illustrated in Supplementary Fig. S1, one approach focuses on environmental assessment using LCA, while the other examines product performance, consumer preferences, or user feedback. LCA studies are useful because they quantify environmental burdens, but they rarely show how alternatives perform in everyday use. In contrast, user-experience studies provide practical information about comfort, durability, and acceptability, but they rarely integrate these findings with per-use GHG evidence in a structured decision-support process. This leaves a significant gap: a material may appear favourable under specific LCA assumptions but still be unacceptable to users, while a popular alternative may not necessarily perform best under the per-use GHG criterion used in the present study.

This gap is relevant to product substitution decisions because favourable per-use GHG performance alone does not establish practical suitability or consumer acceptance. Substitution assessments may therefore benefit from considering the selected climate-related criterion together with reported usability and consumer-approval evidence. Modern data-driven methods, particularly NLP applied to large-scale online customer reviews, enable the extraction of structured information about user perceptions, product performance, and satisfaction. Recent reviews show that NLP has become widely used for analysing online customer reviews across e-commerce settings, with Amazon reviews representing one of the most frequently used data sources in this research area^18^.

In this study, we develop and demonstrate an interpretable AI-enabled decision-support workflow that integrates two complementary evidence domains: (i) literature-derived per-use GHG indicators harmonized from published LCA studies and sustainability reports, and (ii) review-derived user evidence extracted from Amazon customer reviews using NLP. Within the review-derived evidence domain, usability and safety attributes, sentiment patterns, and consumer approval signals were extracted and integrated with the per-use GHG evidence using multi-criteria decision analysis (MCDA). The overall workflow is illustrated in Supplementary Fig. S2, and the taxonomy of evaluated straw materials is shown in Supplementary Fig. S3.

Using drinking-straw alternatives as an information-rich case study, this work addresses three research questions: (1) To what extent are literature-derived per-use GHG indicators and review-derived consumer-approval patterns aligned across straw materials? (2) What usability and sensory patterns are reflected in the review-derived evidence for alternatives that appear favourable under specific LCA assumptions? (3) Can an integrated decision-support workflow produce interpretable rankings under different decision-priority scenarios?

By addressing these questions, this study demonstrates an interpretable AI-enabled decision-support workflow within a clearly defined scope. Rather than developing a new process-based LCA or a comprehensive sustainability assessment, the study integrates literature-derived per-use GHG indicators with review-derived user evidence to support transparent comparison of straw-substitution options within the evaluated dataset and weighting scenarios. The proposed workflow is demonstrated using drinking-straw alternatives and may be adapted and evaluated in future studies involving other product categories, provided that product-appropriate environmental criteria and relevant user-derived evidence are available.

## Materials and methods

### Study design and workflow

This study followed a structured decision-support workflow designed to integrate complementary evidence from two domains: literature-derived per-use GHG indicators and review-derived user evidence. The workflow consisted of five main stages: (i) identification and harmonization of literature-derived per-use GHG indicators for drinking-straw alternatives; (ii) acquisition and preprocessing of online customer reviews; (iii) extraction of sentiment, usability, safety, and consumer-approval features from review-derived evidence; (iv) integration of per-use GHG and user-derived criteria using MCDA under alternative decision-priority scenarios; and (v) interpretable analysis of the resulting rankings using decision-tree modelling and association-rule mining. The extended workflow is summarized in Supplementary Fig. S2.

The study does not perform a new process-based LCA or a comprehensive sustainability assessment. Instead, it uses published LCA studies and sustainability reports as sources of harmonized per-use GHG indicators. Drinking-straw alternatives were selected as an information-rich case study because they are widely discussed in single-use plastic substitution debates, have been examined in prior LCA literature, and generate substantial consumer feedback through online reviews.

### Review-derived dataset acquisition and preprocessing

Amazon customer reviews were collected for drinking-straw products that represent the evaluated material categories. These reviews were treated as review-derived user evidence and as a proxy for user experience, rather than as a representative consumer survey or controlled user-testing dataset. Review acquisition followed a two-stage Python workflow. In the first stage, product names and product-page links were collected from Amazon.co.uk search results and product pages using material-specific search terms corresponding to the straw categories considered in the study. In the second stage, a review-extraction script iterated through the collected product-page links and extracted available customer-review text and associated metadata, including star ratings, timestamps, helpful-vote counts, product labels, and material categories where available. The resulting records were combined into a material-labeled review dataset before text preprocessing. The complete review-derived evidence acquisition and preprocessing workflow, from Amazon product-page acquisition to NLP-based sentiment and feature extraction, is summarized in Supplementary Fig. S4. For consistency and to avoid duplication in website citations, a single representative marketplace source (Amazon.co.uk) was retained for reference reporting in Table 1 and Supplementary Table S1. During preprocessing, duplicate entries, empty reviews, irrelevant records, malformed records, punctuation, and common stop words were removed, and review text was cleaned to improve consistency in downstream analysis. Neutral reviews were retained where relevant for descriptive analyses but were excluded from polarity-based comparisons where classification required a positive or negative orientation.

Table 1 summarizes the review-derived dataset collected across fourteen drinking-straw material categories. In addition to the number of scraped products and reviews, derived descriptive statistics were calculated to indicate review density per product and the relative contribution of each material category to the overall review dataset. Product prices were not included as a comparative criterion because price information was not consistently available in a harmonized form and may vary with seller, package size, marketplace conditions, VAT, shipping, and time of data collection.

**Table 1:** Summary and descriptive statistics of review-derived datasets collected through web scraping for fourteen drinking-straw alternative categories.

No.	Material	No. of scraped products	No. of reviews*	Reviews per product	Share of total reviews (%)
1	Paper	130	882	6.8	19.9
2	Stainless steel	127	992	7.8	22.4
3	Silicone	125	936	7.5	21.1
4	Glass	131	797	6.1	18.0
5	Titanium	57	438	7.7	9.9
6	Avocado	3	31	10.3	0.7
7	PHA	4	41	10.3	0.9
8	Bamboo	13	135	10.4	3.0
9	Aluminium	2	20	10.0	0.5
10	PLA	4	39	9.8	0.9
11	Wheat straws	5	45	9.0	1.0
12	Acrylic	4	37	9.3	0.8
13	Rice straws	1	9	9.0	0.2
14	Sugarcane	1	33	33.0	0.7
	**Total**	**607**	**4435**		**100.0**

Reviews per product was calculated as the number of extracted reviews divided by the number of scraped products in each material category. Share of total reviews represents the percentage contribution of each material category to the full review-derived dataset. Product prices were not used as a decision criterion because price data were not consistently harmonized across products, package sizes, sellers, VAT, shipping conditions, and collection dates.

*Source: Amazon.co.uk product pages; material-specific search-source entries are listed in Supplementary Table S1 and Supplementary References.

### Literature-derived per-use GHG indicators and material screening

The integrated dataset combined two evidence domains. The first domain comprised literature-derived per-use GHG indicators extracted from published LCA studies and sustainability reports reporting climate-related impacts for drinking straw materials. Reported values were harmonized to a per-use basis where necessary to support comparison across materials. The original assumptions, system boundaries, and conversion steps are provided in Supplementary Tables S2a–S2b.

The second domain comprised review-derived user evidence extracted from Amazon customer reviews. The review-derived evidence was used to characterize consumer approval, sentiment, and user-experience features related to usability, safety, durability, comfort, and functionality.

The integrated comparison was restricted to materials for which both evidence domains were available with sufficient consistency. Accordingly, five materials were retained for the final integrated assessment: Paper, Glass, Silicone, Stainless Steel, and Titanium. Conventional plastic straws were not included in the integrated MCDA ranking because equivalent review-derived evidence and harmonized per-use evidence under the same integration criteria were not consistently available within the study design. This absence is acknowledged as a limitation rather than treated as evidence of comparative performance. The per-use GHG indicator values, reusability status, and system-boundary assumptions for the five shortlisted materials are summarized in Table 2.

**Table 2:** Per-use global warming potential (GWP) values, reusability status, and system-boundary assumptions for the five shortlisted straw materials included in the composite assessment.

Material	Reusable	System boundary	Per-use GWP (kg CO₂e/use)
Paper (single use)	No	Cradle-to-gate (reported)	0.141
Glass (reusable)	Yes	Cradle-to-Grave (machine wash)	0.0100
Silicone (reusable)	Yes	Cradle-to-Grave (machine wash)	0.00670
Stainless steel (reusable)	Yes	Cradle-to-Grave (machine wash)	0.0200
Titanium (100% recycled, reusable)	Yes	Cradle-to-gate manufacturing + standardized wash assumption	0.004004*

*Estimated value based on a titanium straw mass of 12 g, 150 lifetime uses, and a per-use washing contribution of 0.00338 kg CO₂e/use. Per-use GWP inputs were derived from Rai et al.^19^, Eleni and Boukouvalas^10^, and IperionX Limited^20^.

### Sentiment analysis and user-experience feature extraction

Customer-review text was analysed to derive sentiment and user-experience features related to reported product use. Polarity scores were obtained using lexicon-based NLP tools, including TextBlob and VADER. These methods were selected because they are transparent, interpretable, and suitable for exploratory analysis of large-scale customer-review datasets. Rather than functioning as black-box predictive models, these tools provide reproducible polarity estimates that support comparison across materials. This binary and aggregated coding was used to support transparent comparison across materials, but it simplified complex user-experience attributes and did not capture the intensity, frequency, or severity of specific usability problems.

Review content was screened for aspect-related signals corresponding to bendability, heat and cold tolerance, mouthfeel, child safety, travel/driver safety, cleaning, durability, comfort, and functionality-related concerns. These attributes were then encoded as binary or aggregated indicators for subsequent comparison across materials. In addition to polarity-based sentiment analysis, descriptive lexical analyses were conducted as supplementary support to identify recurring terms in positive and negative user experiences. For the lexical visualizations, positive-review and negative-review subsets were analysed separately. Bigram-frequency plots were generated after removing material names, generic product terms, and repetitive non-informative expressions. Phrase variants with the same practical meaning were normalized before frequency counting; for example, “Clean Brush”, “Brush Clean”, and “Cleaning Brush” were treated as the same user-experience expression. Word-cloud visualizations and additional descriptive material are reported in Supplementary Fig. S5.

### Multi-criteria decision analysis

The per-use GHG and review-derived evidence streams were integrated using a multi-criteria decision-analysis (MCDA) approach. Three dimensions were included in the composite assessment: (i) GHG-derived performance; (ii) user-experience and safety performance; and (iii) consumer approval. These dimensions were represented by the GHG-derived score, user-experience feature score, and consumer-approval score, respectively, as defined in Sect.  2.6. This integration enabled the joint evaluation of the selected climate-related criterion and review-derived user evidence under alternative decision priorities.

Alternative weighting scenarios were predefined to reflect different decision priorities rather than estimated from the data or interpreted as universal sustainability weights. They were used to examine the sensitivity of material-ranking patterns under plausible decision contexts within a standard weighted-sum MCDA approach. The use of explicit and transparent decision-priority weights follows established multi-criteria decision-analysis practice^21,22^.

The evaluated scenarios comprised a balanced scenario, a GHG-prioritized scenario, a user-experience-prioritized scenario, and a scenario excluding consumer approval. Their purpose was not to identify a universally optimal set of weights, but to assess whether the material-ranking patterns remained consistent across decision priorities relevant to single-use product substitution, procurement, product design, and consumer-facing decision support. The scenario definitions, weight settings, and integrated MCDA score equations are provided in Supplementary Section S4.

### Integrated MCDA score formulation

A transparent weighted-sum MCDA was applied to rank the shortlisted straw materials within the evaluated decision matrix^22^. The per-use GHG indicator was transformed into a normalized GHG-derived score using min–max inversion, so that lower per-use emissions corresponded to higher scores. User-experience and safety attributes extracted from the reviews were encoded as binary indicators, with the presence of a favourable attribute assigned a value of 1 and its absence assigned a value of 0. For each material, these indicators were averaged to generate the user-experience feature score. This binary representation provided a transparent summary of the evaluated attributes but did not capture their intensity, frequency, or severity within individual reviews.

The consumer-approval score was calculated as the proportion of reviews with star ratings of ≥ 4/5. The final integrated MCDA score was then computed by combining the GHG-derived score, user-experience feature score, and consumer-approval score using scenario-specific weights. For each scenario, the weights were non-negative and summed to one. The resulting scores and rankings therefore apply to the five evaluated materials, the selected per-use GHG assumptions, the review-derived evidence, the normalization procedure, and the predefined weighting scenarios.

### Interpretable modelling and association-rule mining

To improve interpretability, a shallow decision-tree regressor was used as a post hoc explanatory tool to derive human-readable rules summarizing variation in the GHG-prioritized integrated MCDA score among the five evaluated materials^23^. The model used the per-use GHG indicator and user-experience feature score as explanatory variables and was constrained to a maximum depth of two to preserve interpretability rather than optimize predictive performance. Consumer approval contributed to the integrated target score but was not entered as a separate explanatory variable in the decision tree. In parallel, association-rule mining was conducted using the Apriori algorithm to explore co-occurrence patterns among the five binary user-experience and safety attributes: bendability, heat/cold tolerance, absence of adverse smell/taste effects, child safety, and driving/travel safety^24^. Support represents the proportion of evaluated materials containing an item combination, confidence represents the conditional proportion in which the consequent occurred when the antecedent was present, and lift compares the observed co-occurrence with that expected under independence. Minimum support and confidence thresholds were set to 0.4 and 0.7, respectively. Given the five-material decision matrix and simplified binary coding, the decision-tree results were treated as post hoc explanatory outputs, while the association-rule results were interpreted descriptively; neither analysis was used for prediction, causal inference, or external generalization. The full decision-tree configuration and association-rule settings are reported in Supplementary Section S4.

## Results

### Integrated evidence base

The integrated evidence base combined literature-derived per-use GHG indicators with review-derived user evidence for drinking-straw alternatives. Fourteen straw-material categories comprising 4,435 reviews were initially considered. The final integrated assessment focused on the five materials for which both evidence domains could be consistently assembled: Paper, Glass, Silicone, Stainless Steel, and Titanium. These five shortlisted categories together accounted for 4,045 review records.

The literature-derived evidence showed substantial variation in per-use GHG indicators across materials, reflecting differences in production processes, reuse assumptions, functional units, and end-of-life conditions. Reusable materials generally showed lower per-use GHG indicators when repeated use was assumed, whereas Paper showed a higher per-use GHG indicator in the harmonized dataset. These differences provided the basis for jointly examining per-use GHG indicators and review-derived user evidence in the subsequent integrated assessment.

As described in the Methods, conventional plastic straws were retained as a contextual baseline but were not included as a scored material in the integrated MCDA ranking because comparable review-derived user-experience and consumer-approval evidence was not consistently available under the same integration criteria. The integrated MCDA therefore focused on alternative straw materials for which both evidence domains were available.

### Sentiment-classification patterns

The sentiment-classification results in Table 3 show that reviews of all five shortlisted straw materials were predominantly classified as positive by both lexicon-based methods. TextBlob classified 3,352 of the 4,045 reviews (82.9%) as positive, whereas VADER classified 2,876 reviews (71.1%) as positive, assigned a substantially larger share to the neutral category, and assigned a smaller share to the negative category. This difference reflects the distinct lexicons and polarity classification rules used by the two methods, particularly for reviews with mixed or less explicit sentiment.

At the material level, Glass and Stainless Steel showed the highest positive shares across both TextBlob and VADER. Silicone also showed a predominantly positive sentiment pattern. Titanium remained predominantly positive but had the highest negative share under VADER (3.7%). Paper was predominantly classified as positive by TextBlob. In contrast, VADER classified 37.5% of Paper reviews as neutral, indicating a less strongly positive sentiment pattern under this method than for the main reusable alternatives.

Overall, the broadly positive and partially overlapping material-level patterns indicate limited discrimination when sentiment classification is considered alone. The sentiment outputs were therefore interpreted as descriptive review-derived evidence and kept distinct from the rating-based consumer-approval score used in the MCDA.

**Table 3:** Classification statistics for Amazon customer reviews categorized as positive, neutral, and negative using TextBlob and VADER. Values are reported as numbers of reviews, with percentages in parentheses.

Material	No. of scraped products	Total reviews (*n*)	TextBlob Positive	TextBlob Neutral	TextBlob Negative	VADER Positive	VADER Neutral	VADER Negative
Paper	130	882	670 (76.0%)	135 (15.3%)	77 (8.7%)	536 (60.8%)	331 (37.5%)	15 (1.7%)
Stainless steel	127	992	852 (85.9%)	75 (7.6%)	65 (6.6%)	733 (73.9%)	246 (24.8%)	13 (1.3%)
Titanium	57	438	349 (79.7%)	50 (11.4%)	39 (8.9%)	308 (70.3%)	114 (26.0%)	16 (3.7%)
Silicone	125	936	796 (85.0%)	71 (7.6%)	69 (7.4%)	685 (73.2%)	229 (24.5%)	22 (2.4%)
Glass	131	797	685 (85.9%)	69 (8.7%)	43 (5.4%)	614 (77.0%)	167 (21.0%)	16 (2.0%)

Note: Percentages were recalculated using each material’s total review count as the denominator. Due to one-decimal rounding, row totals may differ slightly from 100.0%. These sentiment-classification percentages are distinct from the rating-based consumer-approval score used in the MCDA.

### Lexical patterns in positive-review and negative-review subsets

**Fig. 1:**
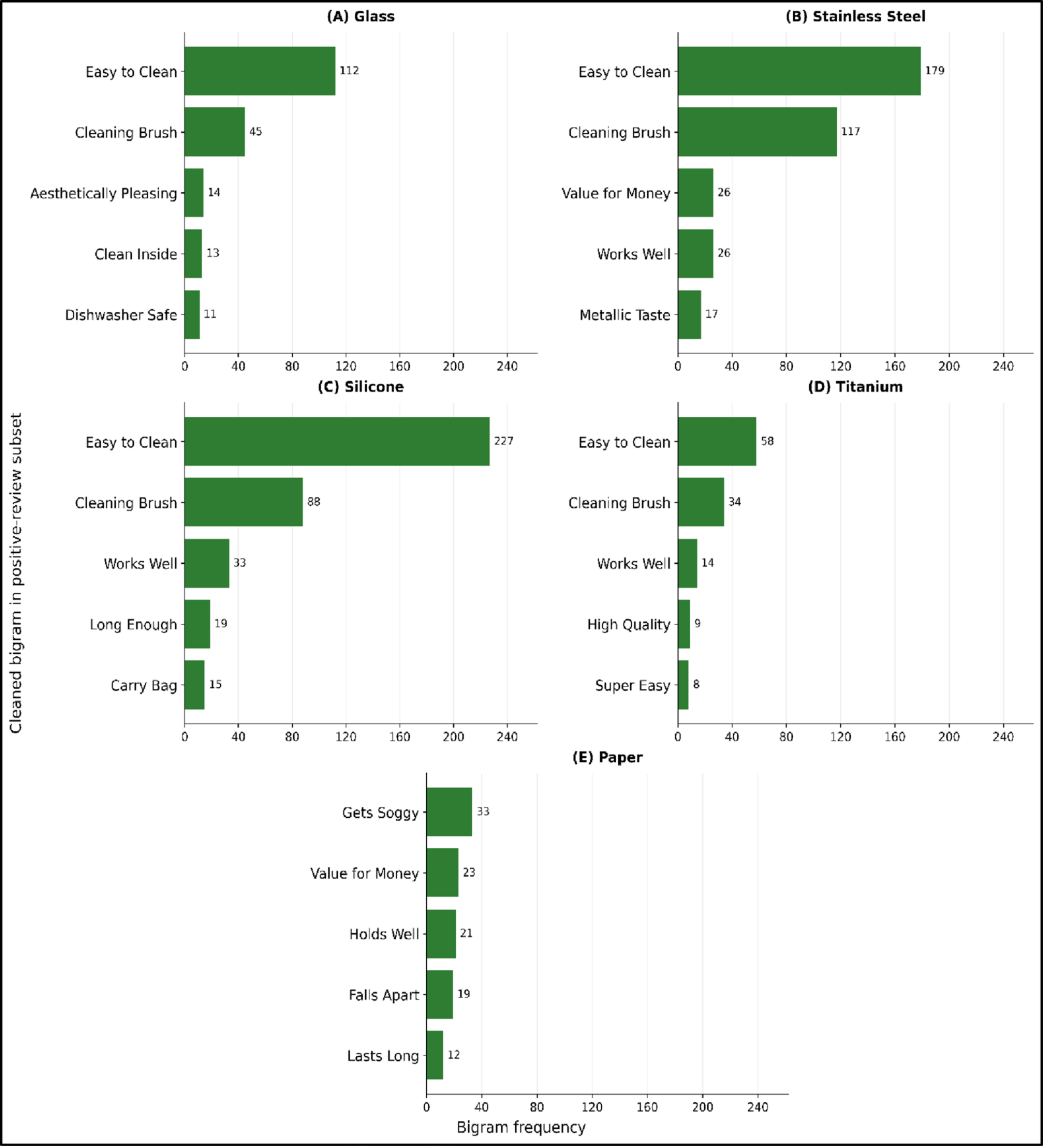
Top-five cleaned informative bigrams identified in the positive-review subset for the five shortlisted straw materials. Generic product terms, material names, brand or product names, repetitive non-informative expressions, and unclear fragments were removed, and phrase variants with the same practical meaning were normalized before frequency counting. The positive-review subset was defined by review-level sentiment classification and does not imply independently positive polarity for every displayed bigram. The corresponding bigrams and frequencies are provided in Supplementary Table S5.

**Fig. 2:**
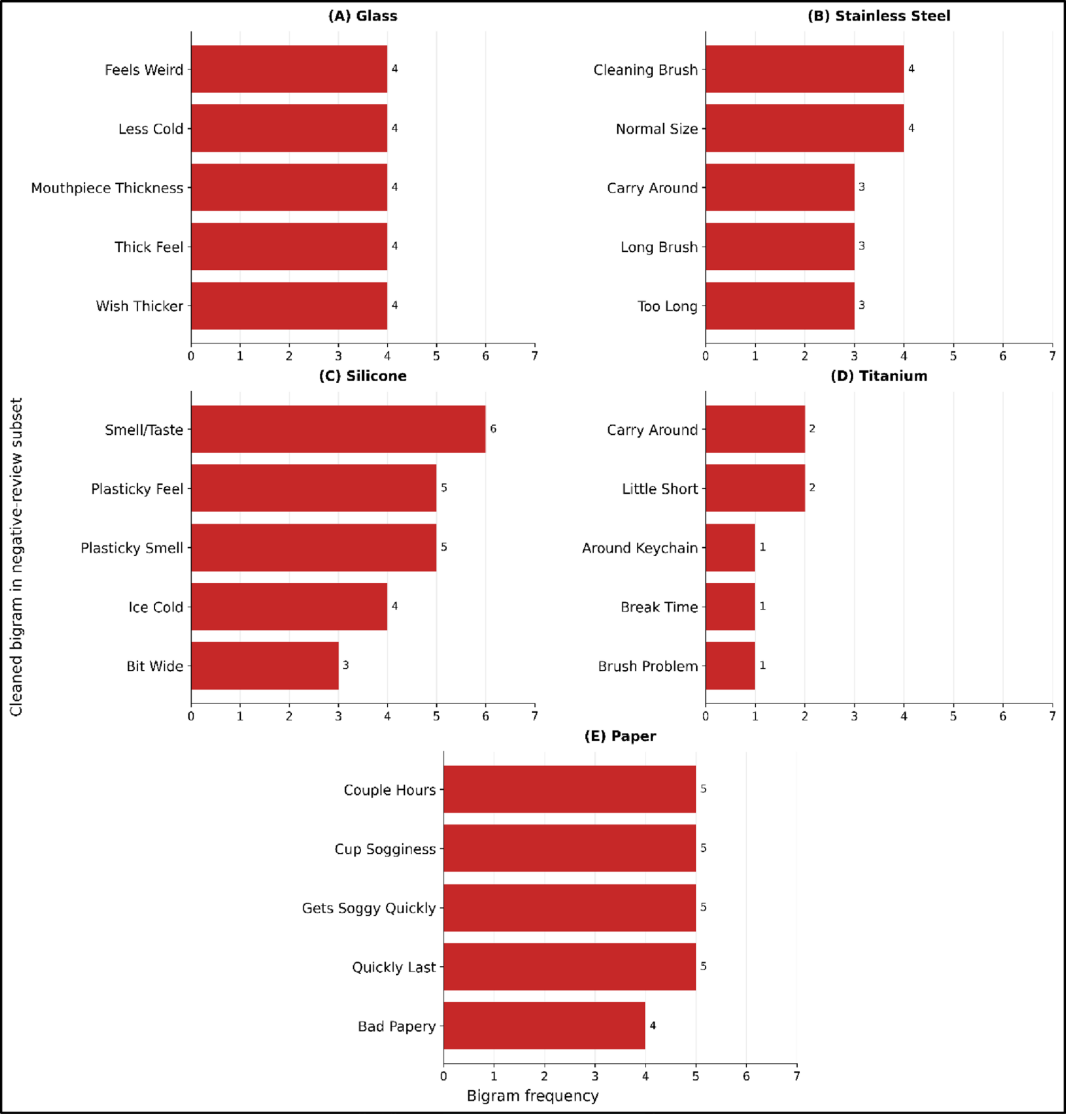
Top-five cleaned informative bigrams identified in the negative-review subset for the five shortlisted straw materials. The same cleaning, phrase-normalization, and frequency-counting procedure used for Fig. 1 was applied. The negative-review subset was defined by review-level sentiment classification and does not imply independently negative polarity for every displayed bigram. The corresponding bigrams and frequencies are provided in Supplementary Table S5.

Figures 1 and 2 present the top-five cleaned informative bigrams identified in the positive-review and negative-review subsets, respectively, for the five shortlisted straw materials. The two subsets were defined using review-level sentiment classification; therefore, the occurrence of a bigram within the positive-review or negative-review subset does not necessarily indicate that the bigram itself has independently positive or negative polarity. Generic product terms, material names, brand or product names, repetitive non-informative expressions, and unclear fragments were removed, and phrase variants with the same practical meaning were normalized before frequency counting. The corresponding bigrams and frequencies are reported in Supplementary Table S5.

Material-level lexical patterns differed across the two review-level sentiment subsets. Cleaning-related expressions recurred for Glass, Stainless Steel, Silicone, and Titanium, while sensory, dimensional, portability, durability, and functionality-related expressions varied among materials. Paper reviews contained recurring expressions related to sogginess and duration of use. These patterns provide descriptive context for the review-derived evidence but do not establish independent sentiment polarity, causal effects on consumer approval, or separate MCDA criteria. Additional descriptive word-cloud visualizations are provided in Supplementary Fig. S5.

### Integrated dataset and derived features

As summarized in Table 4, the integrated per-use GHG, user-experience, and consumer-approval indicators showed distinct profiles across the five shortlisted straw materials. Silicone combined a low per-use GHG indicator (0.0067 kg CO₂e/use) with the highest user-experience feature score (4/5; 80%) and a consumer-approval score of 0.790. Glass also showed a low per-use GHG indicator (0.0100 kg CO₂e/use), together with a moderate user-experience feature score (2/5; 40%) and the highest consumer-approval score among the five materials (0.815). Titanium had the lowest per-use GHG indicator (0.0040 kg CO₂e/use), together with a moderate user-experience feature score and a consumer-approval score of 0.680. Stainless Steel showed a relatively low per-use GHG indicator (0.0200 kg CO₂e/use) and a consumer-approval score of 0.800, but the lowest user-experience feature score among the reusable alternatives (1/5; 20%). Paper had the highest per-use GHG indicator (0.1410 kg CO₂e/use), a moderate user-experience feature score (2/5; 40%), and a consumer-approval score of 0.685. For the subsequent MCDA, the per-use GHG indicator was transformed by min–max inversion to obtain the GHG-derived score, which was then combined with the user-experience feature score and rating-based consumer-approval score under the predefined weighting scenarios.

**Table 4:** Integrated per-use GHG indicator, user-experience feature score, and rating-based consumer-approval score used to construct the MCDA criteria for the five shortlisted straw materials.

Material	Per-use GHG indicator (kg CO₂e/use; lower is better)	User-experience feature score (*n*/5, %)	Consumer-approval score used in MCDA (0–1)
Paper	0.1410	2/5, 40	0.685
Glass	0.0100	2/5, 40	0.815
Silicone	0.0067	4/5, 80	0.790
Stainless steel	0.0200	1/5, 20	0.800
Titanium	0.0040	2/5, 40	0.680

Consumer approval was defined as the share of reviews with star ratings of ≥ 4/5 and is reported separately from the TextBlob and VADER sentiment-classification percentages shown in Table 3. The user-experience feature score was based on five binary favourable attributes and therefore does not represent the intensity, frequency, or severity of reported user-experience concerns.

### Multi-criteria ranking and interpretability

**Fig. 3:**
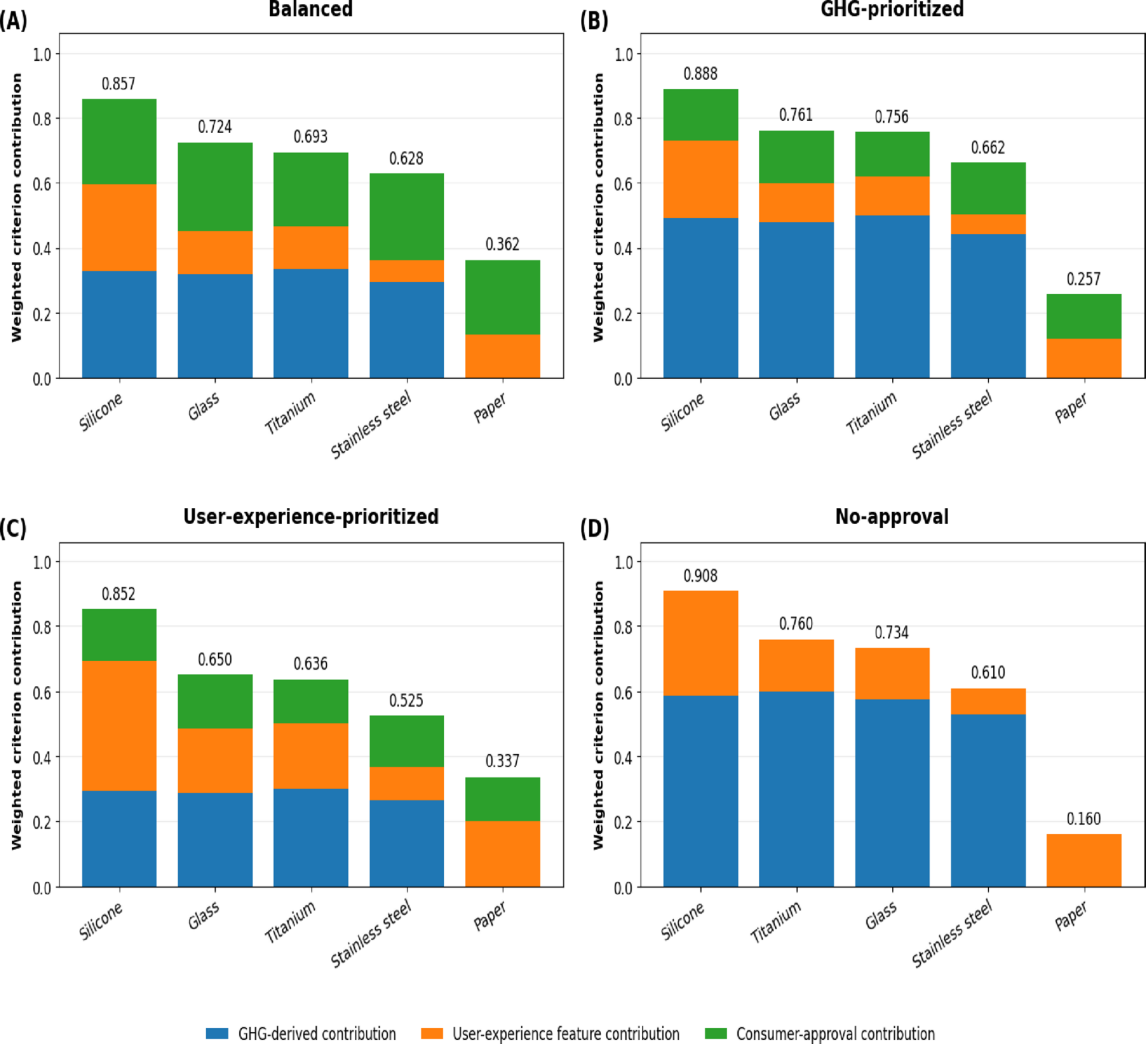
Weighted criterion contributions and integrated MCDA scores for the five shortlisted straw materials under four predefined decision-priority scenarios: (**A**) balanced, (**B**) GHG-prioritized, (**C**) user-experience-prioritized, and (**D**) no-approval. Each stacked bar shows the weighted contributions of the GHG-derived score, user-experience feature score, and rating-based consumer-approval score, where applicable. The numerical label above each bar represents the resulting integrated MCDA score within the evaluated five-material decision matrix under the corresponding scenario-specific weights.

Using the predefined weighting scenarios and scenario-specific weighted-sum equations detailed in Supplementary S4, an integrated MCDA score was calculated for each shortlisted material. The score combined three components: the GHG-derived score obtained through min–max inversion of the per-use GHG indicator, the user-experience feature score calculated from the five binary usability and safety attributes, and the rating-based consumer-approval score. Figure 3 presents the weighted criterion contributions and resulting integrated MCDA scores under the four predefined decision-priority scenarios.

Among the five shortlisted materials, Silicone achieved the highest integrated MCDA score in all four scenarios, whereas Paper ranked lowest. These results are conditional on the evaluated dataset, selected per-use GHG inputs and harmonization assumptions, available review-derived user evidence, min–max normalization procedure, and scenario-specific weights. Under the balanced scenario, Silicone achieved an integrated MCDA score of 0.857, followed by Glass (0.724), Titanium (0.693), Stainless Steel (0.628), and Paper (0.362).

Glass ranked second under the balanced, GHG-prioritized, and user-experience-prioritized scenarios, whereas Titanium moved to second place when consumer approval was excluded. Stainless Steel remained fourth and Paper remained fifth across all four scenarios. These results indicate that the highest and lowest ranking positions were stable within the evaluated scenarios, while the relative ordering of Glass and Titanium was sensitive to the inclusion and weighting of consumer approval.

**Fig. 4:**
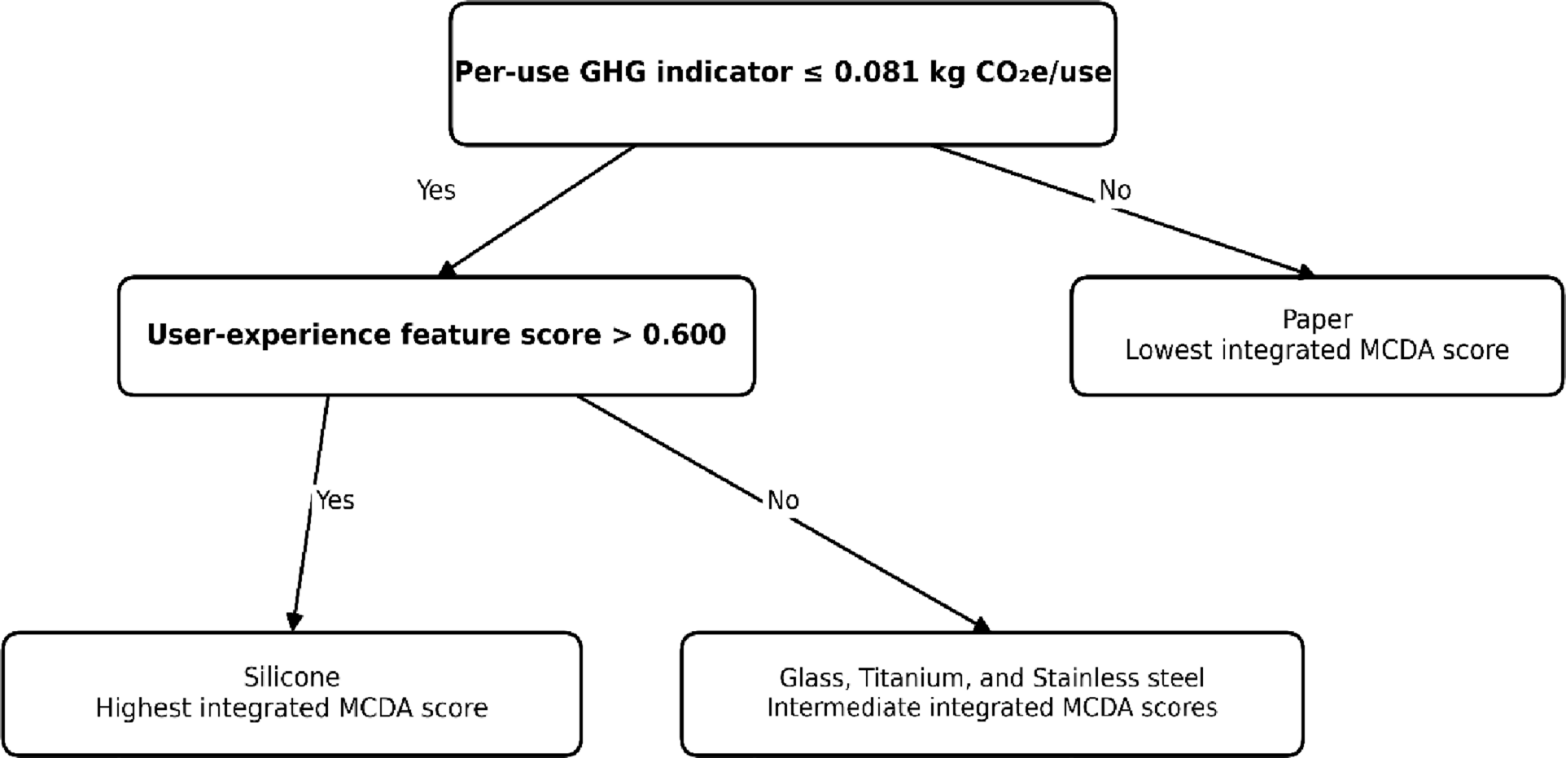
Post hoc shallow decision-tree explanation of the GHG-prioritized integrated MCDA scores within the evaluated five-material decision matrix. The tree used the per-use GHG indicator and user-experience feature score as explanatory variables. Consumer approval contributed to the integrated target score but was not entered as a separate explanatory variable. The displayed thresholds are internal explanatory splits and are not universal environmental or usability benchmarks or independently validated predictive thresholds.

To enhance interpretability of the ranking results, Fig. 4 presents a post hoc shallow decision-tree explanation of the GHG-prioritized integrated MCDA scores. The tree used the per-use GHG indicator and user-experience feature score as explanatory variables. Consumer approval contributed to the integrated target score but was not supplied as a separate explanatory variable. The first split, at a per-use GHG indicator of 0.081 kg CO₂e/use, separated Paper from the four reusable alternatives within the evaluated dataset. Within the lower-per-use-GHG group, a user-experience feature-score threshold of 0.600 separated Silicone from Glass, Titanium, and Stainless Steel. These thresholds are internal explanatory splits derived from the five-material decision matrix and should not be interpreted as universal environmental or usability benchmarks or as independently validated predictive thresholds.

### Exploratory association-rule analysis

Apriori association-rule mining was applied as an exploratory descriptive analysis of the five binary user-experience and safety attributes: bendability, heat/cold tolerance, absence of adverse smell/taste effects, child safety, and driving/travel safety. Two directional rules met the predefined minimum support and confidence thresholds: child safety → driving/travel safety and driving/travel safety → child safety. Each rule had support = 0.40, confidence = 1.00, and lift = 2.50. Because the analysis included five materials, support = 0.40 indicates that the attribute combination occurred in two materials. Given the five-material decision matrix and simplified binary coding, these rules were interpreted only as exploratory descriptive co-occurrence patterns. They were not used for prediction, causal inference, material ranking, consumer-approval conclusions, or modification of the MCDA scores. The full association-rule settings and results are reported in Supplementary S4.

## Discussion

### Integrating complementary evidence supports contextualized substitution assessment

Within the evaluated five-material dataset, literature-derived per-use GHG indicators and review-derived user evidence did not produce fully aligned material profiles. Materials with lower per-use GHG indicators did not necessarily achieve the highest user-experience feature or consumer-approval scores, while highly rated materials did not always show the lowest per-use GHG indicators. This lack of complete alignment indicates that no single evaluated criterion was sufficient to characterize the substitution options considered in this study.

The proposed decision-support workflow provided a structured approach for examining this lack of alignment by integrating two complementary evidence domains: literature-derived per-use GHG indicators and review-derived user evidence. The workflow does not replace process-based life-cycle assessment, controlled usability testing, or comprehensive sustainability assessment. Instead, it provides a transparent structure for comparing material alternatives within the selected criteria, harmonization assumptions, and scenario-specific weights. The review-derived evidence should therefore be interpreted as a proxy for reported user experience and consumer approval rather than as representative evidence of population-wide preferences.

The integrated results illustrate how combining climate-related and user-oriented evidence can reveal trade-offs that would remain less visible if either evidence domain were considered independently. This interpretation is restricted to the evaluated materials, the selected per-use GHG assumptions, the available review-derived evidence, and the predefined MCDA scenarios. Accordingly, the resulting rankings should be understood as contextual decision-support outputs rather than general judgments about the overall sustainability of the materials.

### Review-derived user evidence contextualizes practical suitability

The review-derived analyses showed that practical product attributes differed across the evaluated straw materials. The lexical and sentiment analyses identified recurring expressions related to cleaning, comfort, sensory experience, dimensions, portability, durability, and duration of use. These patterns provide descriptive context for how users discussed the products, but they should not be interpreted as direct measures of adoption or as evidence that attributes caused higher or lower consumer approval.

The results nevertheless indicate that per-use GHG indicators alone do not capture all dimensions relevant to practical substitution. Materials with favourable climate-related indicators may still be associated with usability or sensory concerns, while materials with higher consumer-approval scores may not have the lowest per-use GHG indicators. Review-derived user evidence therefore provided a complementary proxy for reported product experience within the available Amazon review dataset. It does not replace representative consumer surveys, controlled usability testing, or direct observation of long-term product use.

These descriptive patterns are broadly consistent with previous studies showing that practical performance and consumer response are relevant when evaluating straw alternatives. Liang et al.^15^ reported that ease of straw insertion and fracture affected purchase willingness in controlled consumer testing, while Gutierrez et al.^16^ documented substantial loss of paper-straw compressive strength after immersion. Paliwoda et al.^17^ further showed that environmental indicators can influence purchasing decisions for lifestyle products. The present review-derived findings complement, but do not replace, such controlled testing and survey evidence.

The post hoc shallow decision-tree analysis further supported interpretation of the integrated MCDA outputs by summarizing how the per-use GHG indicator and user-experience feature score separated higher, intermediate, and lower GHG-prioritized integrated MCDA scores within the five-material decision matrix. Consumer approval contributed to the integrated target score but was not entered as a separate explanatory variable. The tree was used as an explanatory representation of the calculated MCDA results rather than as a predictive model of consumer behaviour, market adoption, or material performance. Its thresholds are specific to the evaluated materials, input values, normalization procedure, and GHG-prioritized scenario and should not be treated as general product-design, environmental, or usability benchmarks.

### Decision-support implications

The principal value of the proposed workflow lies in supporting transparent comparison among substitution options when multiple evidence sources must be considered simultaneously. Within the present case study, the evaluated alternatives showed trade-offs among literature-derived per-use GHG indicators, user-experience features, and rating-based consumer approval. Considering only one criterion could therefore favour an option that performs well on that dimension while overlooking limitations identified through the other evaluated evidence streams.

In environmental-management and procurement contexts, the workflow may serve as an exploratory screening aid for structuring comparisons before more detailed assessment or implementation. It can help decision-makers identify how rankings change when greater emphasis is placed on climate-related performance, user-experience features, or consumer approval. However, the outputs should not be used as stand-alone procurement recommendations or policy prescriptions without context-specific data, stakeholder-defined priorities, and additional environmental and usability evidence.

The workflow also improves transparency by displaying the contribution of each criterion to the integrated MCDA score and by examining the sensitivity of rankings across predefined weighting scenarios. This makes the basis of the comparison more explicit than a single aggregated recommendation. The resulting rankings remain conditional on the selected materials, harmonized per-use GHG indicators, review-derived user evidence, normalization choices, and scenario-specific weights.

Although demonstrated using drinking-straw alternatives, the workflow may be adapted to other product-substitution contexts when comparable environmental and user-oriented evidence can be assembled. Such applications require product-specific criteria, appropriate evidence harmonization, clearly justified weights, and independent validation before operational use.

### Study limitations and future directions

Several limitations should be considered when interpreting the findings. First, the environmental evidence was restricted to literature-derived per-use GHG indicators harmonized from previously published studies rather than generated through a new process-based life-cycle assessment. Although harmonization enabled comparison on a common per-use basis, differences in original functional units, system boundaries, reuse assumptions, washing conditions, transport, durability, and end-of-life scenarios could not be fully eliminated. The assessment therefore represents a climate-related indicator comparison and should not be interpreted as a comprehensive sustainability assessment.

Second, the analysis did not include other potentially relevant environmental dimensions, such as resource depletion, toxicity, water use, littering potential, persistence, recyclability, compostability, or leakage into terrestrial and marine environments. The relative rankings among materials may change if these dimensions are incorporated. Future studies should therefore extend the environmental evidence base using product-specific criteria and, where feasible, consistently modelled life-cycle inventories.

Third, the user-oriented evidence was derived from Amazon customer reviews and should be interpreted as review-derived proxy evidence rather than as a representative consumer survey or controlled usability assessment. Online reviews may be affected by self-selection, platform-specific user populations, product availability, reviewer expectations, and differences among individual products grouped within the same material category. The rating-based consumer-approval score and lexicon-based sentiment classifications also capture different aspects of the review evidence and should not be treated as interchangeable measures.

Fourth, the user-experience feature score was based on simplified binary coding of five usability and safety attributes. This representation facilitated transparent integration within the MCDA but did not capture the intensity, frequency, severity, or context of reported product attributes. Similarly, lexical frequencies describe recurring expressions within review-level sentiment subsets but do not establish the independent polarity, prevalence among all users, or causal effect of individual product characteristics.

Fifth, the integrated MCDA was based on a five-material decision matrix, min–max normalization, and four predefined weighting scenarios. The resulting scores and rankings are therefore conditional on the evaluated materials, selected per-use GHG assumptions, available review-derived evidence, normalization procedure, and scenario-specific weights. Weights were used to examine sensitivity to different decision priorities and should not be interpreted as universal sustainability preferences. Broader applications should incorporate stakeholder-defined weights, alternative normalization procedures, uncertainty and sensitivity analyses, and validation using additional product categories and datasets.

The shallow decision tree and Apriori association-rule analysis were constrained by the five-material decision matrix and simplified binary feature coding. Accordingly, the decision-tree thresholds and association rules should be interpreted only as internal explanatory and exploratory descriptive patterns, respectively, rather than as independently validated or externally generalizable predictive, causal, environmental, usability, product-design, or consumer-behaviour relationships.

Finally, conventional plastic straws were retained as a contextual baseline but were not included as a scored alternative in the integrated MCDA because comparable review-derived user-experience and consumer-approval evidence was not consistently available under the same inclusion criteria. Their exclusion limits direct comparison between conventional plastic and the evaluated alternatives. Future research could address this limitation by assembling fully harmonized environmental and user-oriented evidence for the baseline product and by expanding the analysis to include additional materials, platforms, user groups, and product-use contexts.

Future studies should also examine uncertainty propagation during evidence harmonization, test alternative MCDA methods, and evaluate the workflow prospectively in procurement, product-design, or policy contexts. Such extensions would help determine whether the workflow remains informative when applied to larger decision matrices and independently collected environmental and user-experience data.

## Conclusions

This study developed and demonstrated an interpretable AI-enabled decision-support workflow that integrates literature-derived per-use GHG indicators with review-derived user evidence for the comparative assessment of drinking-straw alternatives. The workflow combines a GHG-derived score, a simplified user-experience feature score, and a rating-based consumer-approval score within a transparent MCDA structure and examines the sensitivity of the resulting rankings across predefined decision-priority scenarios.

Among the five shortlisted materials, Silicone achieved the highest integrated MCDA score across all four scenarios, whereas Paper ranked lowest. These results are conditional on the evaluated dataset, selected per-use GHG inputs and harmonization assumptions, available review-derived user evidence, normalization procedure, and scenario-specific weights. Glass ranked second in three scenarios, while Titanium moved to second place when consumer approval was excluded. The highest and lowest ranking positions were therefore stable within the evaluated scenarios, whereas the relative ordering of some intermediate alternatives remained sensitive to the inclusion and weighting of consumer approval.

The main contribution of the study is not a new process-based life-cycle assessment or a comprehensive sustainability ranking of straw materials. Rather, it is the demonstration of a transparent workflow for integrating climate-related and review-derived user evidence while retaining visibility of individual criterion contributions, weighting assumptions, and ranking sensitivity. The review-derived evidence should be interpreted as a proxy for reported user experience and consumer approval rather than as representative evidence of population-wide preferences, controlled product performance, or long-term adoption.

The resulting rankings are contextual decision-support outputs and should not be interpreted as universal judgments about the overall sustainability of the evaluated materials. Future research should evaluate the workflow using larger material sets, additional environmental indicators, independently collected usability evidence, stakeholder-defined weights, and explicit uncertainty analysis. Such extensions would help determine its applicability to broader product-substitution, procurement, and environmental-management decisions.

## Supplementary Information

Below is the link to the electronic supplementary material.

Supplementary Material 1.

Supplementary Material 2.

## Data Availability

The processed dataset supporting the findings of this study is provided as Supplementary Material. All other relevant data are included in the article and its Supplementary Information.

## Code availability

The custom Python scripts used for review preprocessing, sentiment analysis, user-experience feature extraction, MCDA scoring, decision-tree modelling, association-rule mining, and lexical visualization are deposited in Zenodo at 10.5281/zenodo.21432024.
