# Supplementary material for: Interpretable AI-enabled decision support for drinking-straw substitution using per-use greenhouse-gas indicators and user-review evidence
Source: Sci Rep. 2026 Jul 29;16:23558. doi: 10.1038/s41598-026-63847-8 (PMC13421661; doi:10.1038/s41598-026-63847-8)
Supplement: Supplementary file 1 — Supplementary Material 1. [file 41598_2026_63847_MOESM1_ESM.docx]

# Supplementary Information

to the article "**Interpretable AI-enabled decision support for drinking-straw substitution using per-use greenhouse-gas indicators and user-review evidence**".

**(Hassan et al.)**

**Abbreviations:** LCA = life-cycle assessment; FU = functional unit; CtG = cradle-to-gate; C2G = cradle-to-grave; GtG = gate-to-grave; EoL = end-of-life; GHG = greenhouse gas; GWP = global warming potential; CO₂e = carbon dioxide equivalent; MCDA = multi-criteria decision analysis; NLP = natural language processing.

## Supplementary S1. Straw alternatives: materials, advantages and limitations, typical uses, and references

| **Table S1**. Straw alternatives: materials, advantages and limitations, typical uses, and references. | | | | | |
| --- | --- | --- | --- | --- | --- |
| **Type of straw** | **Material** | **Advantages** | **Limitations** | **Typical uses** | **References** |
| 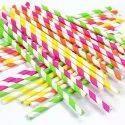Paper | Paper | Biodegradable; inexpensive. | Soggy after prolonged use; not suitable for hot drinks. | Single-use beverages; eco-friendly events. | [25] |
| 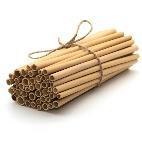Bamboo | Natural bamboo | Reusable; biodegradable. | Requires cleaning; can split over time. | Eco-friendly restaurants; home reuse. | [26] |
| Glass  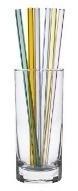 | Tempered glass | Reusable; elegant; easy to clean. | Breakable; not ideal for children. | Home & upscale venues. | [27] |
| 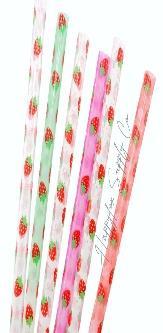Acrylic | PMMA | Rigid; reusable. | Not heat-tolerant; micro-scratch. | Bars; reusables. | [28] |
| 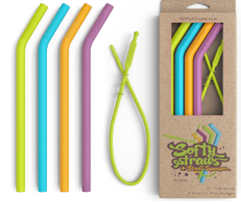Silicone | Food-grade silicone | Flexible; safe for children; heat/cold tolerant. | May retain odours if not cleaned well. | Kids; travel; repeated household use. | [29] |
| 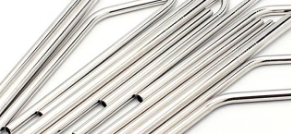Aluminium | Aluminium | Lightweight; recyclable. | Mouthfeel; dentable. | Travel kits; reusable. | [30] |
| 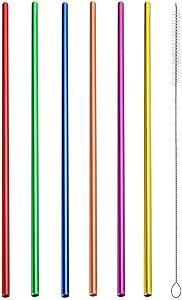Stainless steel | 304/316 Stainless steel | Durable; reusable; recyclable. | Metallic mouth feel; safety in driving contexts. | Cafés; bars; long-term reuse. | [31] |
| 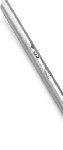Titanium | Recycled Ti (where available) | Lightweight; durable; low GWP (with recycled stock). | Higher purchase cost; limited LCA reporting. | Travel; premium reusable kits. | [32] |
| PLA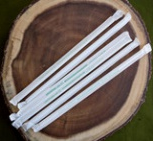 | Polylactic acid | Bio-based; familiar processing. | Compostability limits; heat deformation. | Fast-service; cold drinks. | [33] |
| 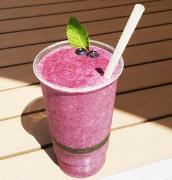PHA | Polyhydroxyalkanoates | Biodegradable (industrial). | Facility-dependent; cost. | Regulated venues. | [34] |
| 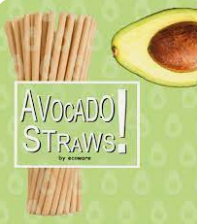Avocado | Agro-residue | Bio-based; compostable in settings. | Performance variability; moisture sensitivity. | Events; niche brands. | [35] |
| 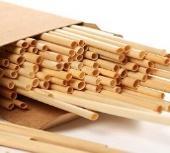Wheat straws | Agricultural residue | Low-cost; rustic aesthetic. | Fragility; taste transfer possible. | Events; local markets. | [36] |
| Rice straws  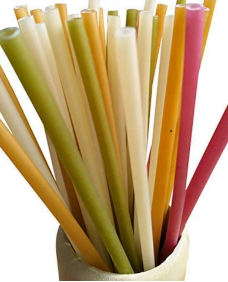 | Cellulose–silica composite | Good wet strength; lower CF in scenarios. | Early stage; limited availability. | Pilots; research products. | [37] |
| 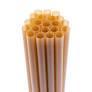Sugarcane | Bagasse | Bio-based; paper-like. | Sogginess; adhesive content issues. | Events; single use. | [38] |

| **Table S2a. Comparative LCA studies on drinking straws**: original functional units, system boundaries, and key qualitative findings. | | | | | |
| --- | --- | --- | --- | --- | --- |
| **Reference** | **Year** | **Country** | **Study scope/objective** | **System boundary** | **Key qualitative finding** |
| [39] | 2014 | Thailand | Carbon footprint assessment of PP vs PBS/PLA blends. | Gate-to-gate /  CF-based | Bioplastics had a higher manufacturing carbon footprint mainly due to waste from impact testing; electricity use was lower than that of PP. |
| [40] | 2020 | Brazil | LCA of six straw materials: steel, glass, paper, jute, plastic, and bamboo. | Cradle-to-grave | Reuse kit design and washing assumptions strongly influence reusable options; proper collection and disposal are critical for single-use items. |
| [41] | 2020 | South Africa | Comparative LCA of disposable and reusable straw options: PP, paper, PLA, glass, and steel. | Cradle-to-grave | The paper showed the lowest GHG impact; glass becomes preferable to steel only after sufficient reuse. |
| [42] | 2021 | Malaysia | Comparative LCA of paper vs bioplastic PLA straws. | Gate-to-grave | PLA showed a lower environmental burden than paper under study-specific assumptions. |
| [43] | 2022 | USA | Comparative LCA of PP, PLA, and paper straws with policy sensitivity, including marine litter. | Production-use-end-of-life | No universal winner was identified; closed-loop recycling substantially reduces PP straw impacts. |
| [44] | 2023 | China | Multi-category LCA of PP, paper, and PLA straws. | Comparative LCA | Results are context-dependent; electricity mix and end-of-life dominate, with no single alternative best across all categories. |
| [45] | 2024 | China/UK | Evaluation of PLA-coated cellulose paper straws with degradation and LCA components | Production-use-disposal | Partial degradation was observed; LCA indicates trade-offs versus conventional options depending on coating and disposal route. |
| [46] | 2024 | India | Development of cellulose-silica straws from rice waste with performance and LCA assessment | Production-use-disposal | Biomass-derived straws showed strong performance and a lower carbon footprint relative to metal/PLA in study scenarios. |
| [47] | 2025 | Greece | Environmental and cost comparison of PP, paper/bioplastics, and reusable straws | Cradle-to-grave, including washing | Dishwashing dominates impacts for metal/glass options; benefits depend on reuse cycles and washing method. |

## Supplementary S2. LCA details and harmonization

***Note****: CF = carbon footprint; PP = polypropylene; PLA = polylactic acid; PBS = polybutylene succinate. System-boundary labels were harmonized from the reviewed studies for compact presentation.*

**Overview of source assumptions and harmonization steps**

The per-use GWP values were derived by aligning reported environmental metrics from different sources as far as possible, while preserving the original assumptions and system-boundary information. For the Greece study, annual impact values were normalized per use, assuming 50 uses per year. The reported dominance of dish washing impacts supported the washing-use assumption applied to reusable materials [47]. For the Thailand study, reported per-kg carbon-footprint values were converted to per-straw values using the 1.33 g/straw functional unit reported in the Malaysia study, and then normalized to kg CO₂e/use according to lifetime-use assumptions [39,42]. For Paper, the 0.141 kg CO₂e/straw value reported by Rai et al. [46] for cradle-to-gate single-straw production was retained as the selected high value for sensitivity/outlier tracking. For titanium, production impacts were contextualized using energy-intensity evidence and a recycled titanium emission factor, while the washing component was harmonized using the reusable-material washing assumption [47–49].

Only the five materials with both literature-derived per-use GHG indicators and sufficient Amazon review volume were retained for the integrated MCDA comparison. The notes below document the harmonization logic and the titanium-specific assumption used in Supplementary Table S2b.

**Method notes: Titanium per-use GWP calculation**

No peer-reviewed cradle-to-grave GWP value specific to titanium drinking straws was identified. Titanium was therefore estimated using a recycled titanium production factor and a harmonized washing-use assumption. Titanium production is known to be energy-intensive [48]. As an indicative factor for secondary titanium, IperionX Limited [49] reports 7.8 kg CO₂e/kg for 100% recycled spherical titanium powder. For reusable materials, washing can dominate the impacts, accounting for approximately 85% of the impacts for metal/glass options in the Greek household LCA by Eleni and Boukouvalas [47].

**Equations**

The equations below follow a standard life-cycle accounting approach in which manufacturing and use-phase contributions are aggregated and normalized per functional use. The equations are used only as calculation steps; parameter values are either literature-derived or explicitly stated assumptions.

Manufacturing contribution:
**GWP_ctg = m × EF_Ti**

Use-phase washing over lifetime:
**GWP_wash,life = N × ω × EF_wash**

Per-use footprint:
**GWP_per-use = (GWP_ctg + GWP_wash,life) / N**

Numerical example used for titanium in Supplementary Table S2b:
m = 0.012 kg; EF_Ti(recycled) = 7.8 kg CO₂e/kg [49]; N = 150 uses; ω = 1 wash/use; EF_wash = 0.00338 kg CO₂e/wash, harmonized from Eleni and Boukouvalas [47].

**GWP_per-use = [(7.8 × 0.012) + (150 × 1 × 0.00338)] / 150 ≈ 0.004004 kg CO₂e/use.**

Symbols: m = straw mass (kg); EF_Ti = cradle-to-gate emission factor for titanium metal (kg CO₂e/kg); N = lifetime uses; ω = washes per use; EF_wash = CO₂e per wash.

Consistency check: washing share = 0.00338/0.004004 ≈ 84.5%, which aligns with the approximately 85% dominance observed for reusable metal/glass options by Eleni and Boukouvalas [47].

| **Table S2b.** Harmonized per-use GWP for materials entering the integrated assessment (n = 5), with conversion factors and assumptions. | | | | | | | |
| --- | --- | --- | --- | --- | --- | --- | --- |
| **Reference** | **Material** | **Reusable** | **Original FU**  **(as published)** | **Reported value (units)** | **Conversion to per-use** | **Per-use GWP (kg CO₂e/use)** | **Assumptions/Notes** |
| [46] | Paper  (single-use) | No | Per straw (CtG) | 0.141 kg CO₂e/straw | per-use = reported | 0.141000 | As reported in the source, retained as a high paper value for sensitivity/outlier tracking. |
| [47] | Glass | Yes | Per use (machine wash) | 0.0100 kg CO₂e/use | per-use = reported | 0.010000 | Greek household LCA; dishwasher load as in source. |
| [47] | Silicone | Yes | Per use (machine wash) | 0.0067 kg CO₂e/use | per-use = reported | 0.006700 | Greek household LCA; dishwasher load as in source. |
| [47] | Stainless steel | Yes | Per use (machine wash) | ≈0.0200 kg CO₂e/use | per-use = reported/approximated | 0.020000 | Approximate per-use value from published figure; washing assumptions harmonized. |
| [47,49] | Titanium (100% recycled) | Yes | kg Ti powder (CtG) + wash-use | 7.8 kg CO₂e/kg | (7.8 × 0.012)/150 + 0.00338 | 0.004004 | m = 12 g; N = 150 uses; EF_wash = 0.00338 kg CO₂e/use; unified washing assumption. |

***Footnote S2b:*** *FU = functional unit; CtG = cradle-to-gate; GWP = global warming potential; CO₂e = carbon dioxide equivalent; MCDA = multi-criteria decision analysis. Only the five materials with both literature-derived per-use GHG indicators and sufficient Amazon review volume entered the integrated assessment*.


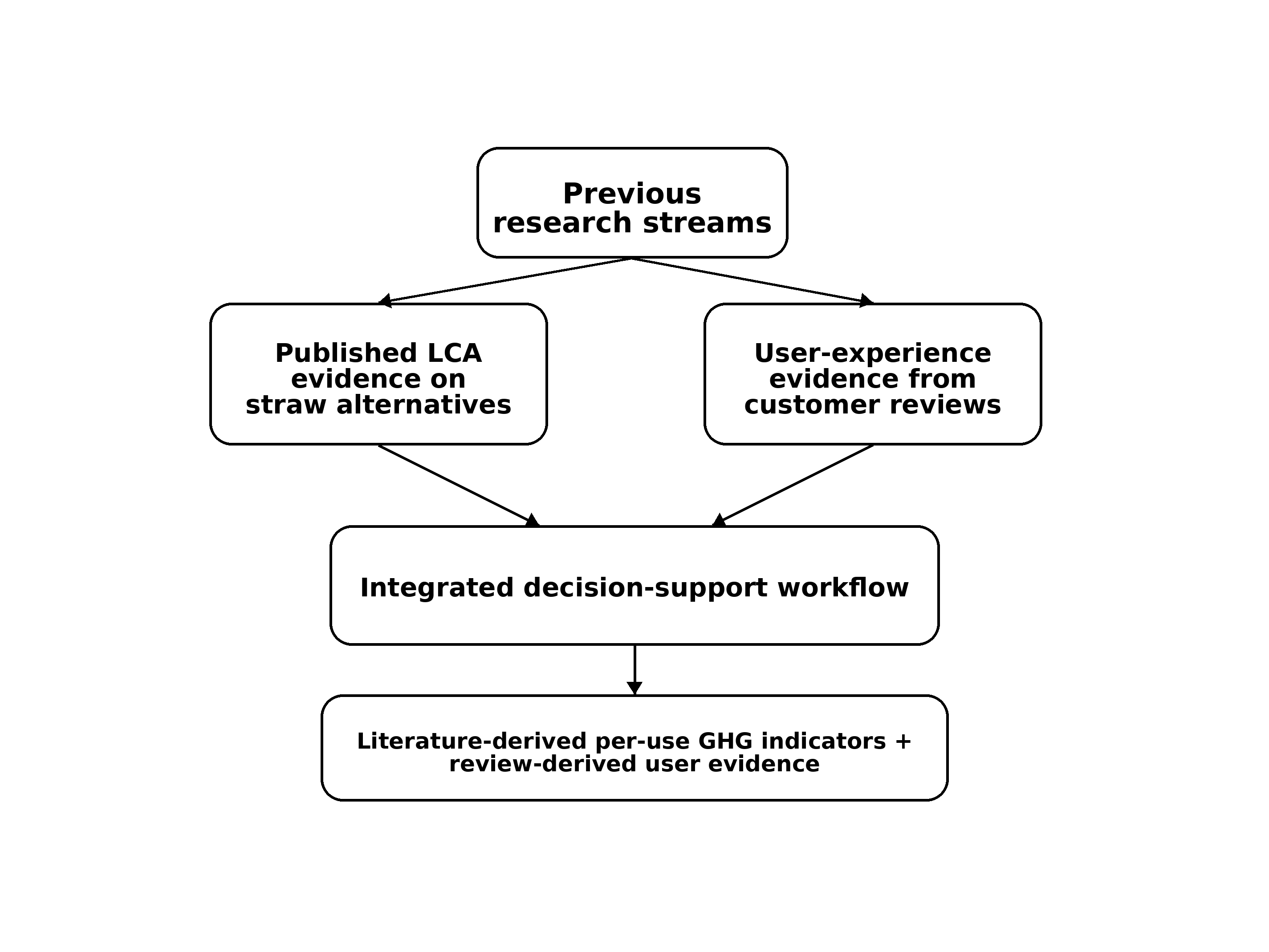


**Supplementary Fig. S1. Main research streams on drinking-straw alternatives and the proposed integrated decision-support workflow.** Previous studies have mainly focused on either published life-cycle assessment evidence or review-derived user evidence. The present study integrates literature-derived per-use GHG indicators with review-derived user evidence within a structured decision-support workflow**.**


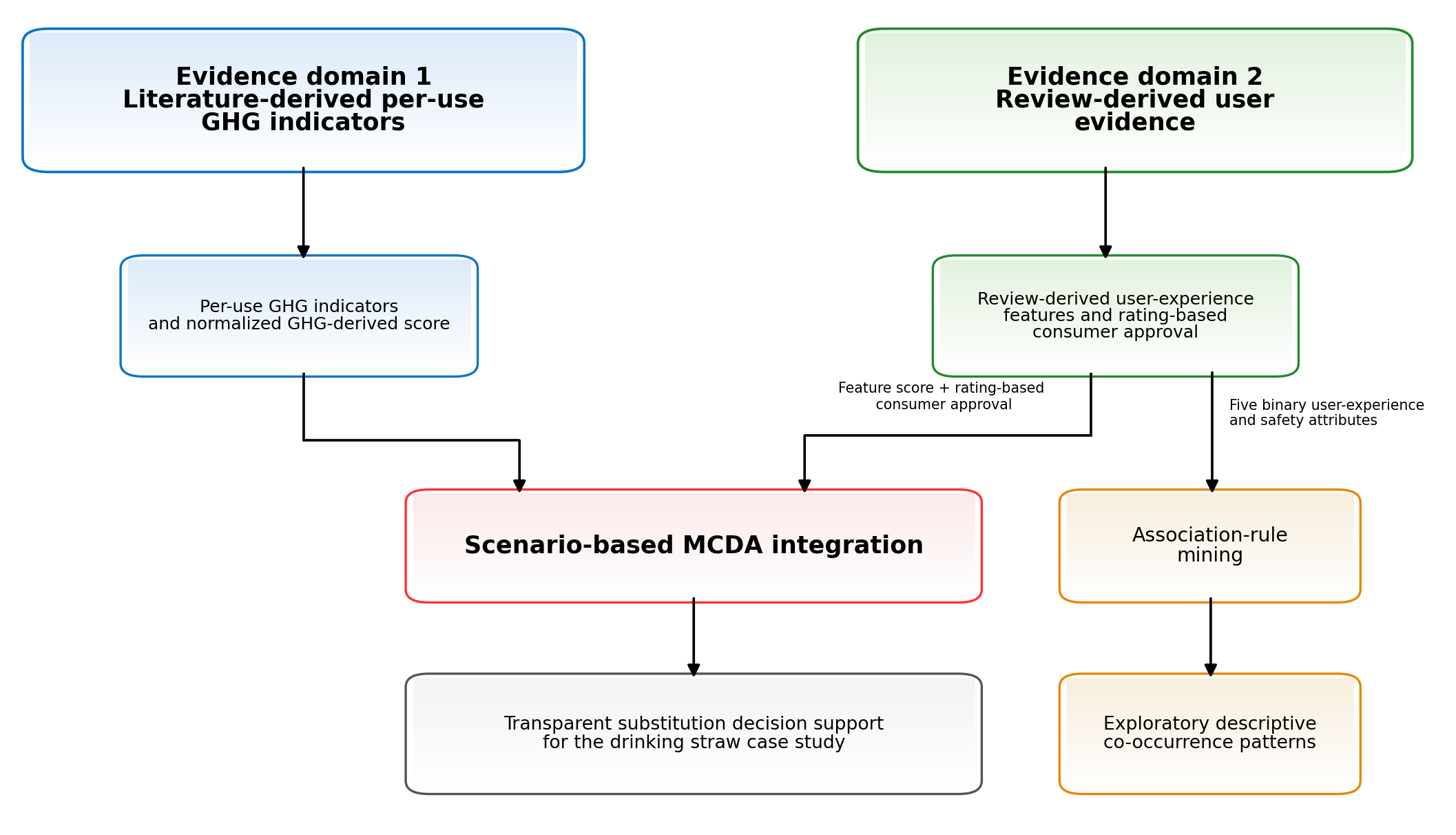
**Supplementary Fig. S2**. Integrated decision-support workflow combining literature-derived per-use GHG evidence with Amazon review-derived user evidence for the evaluation of drinking-straw alternatives.


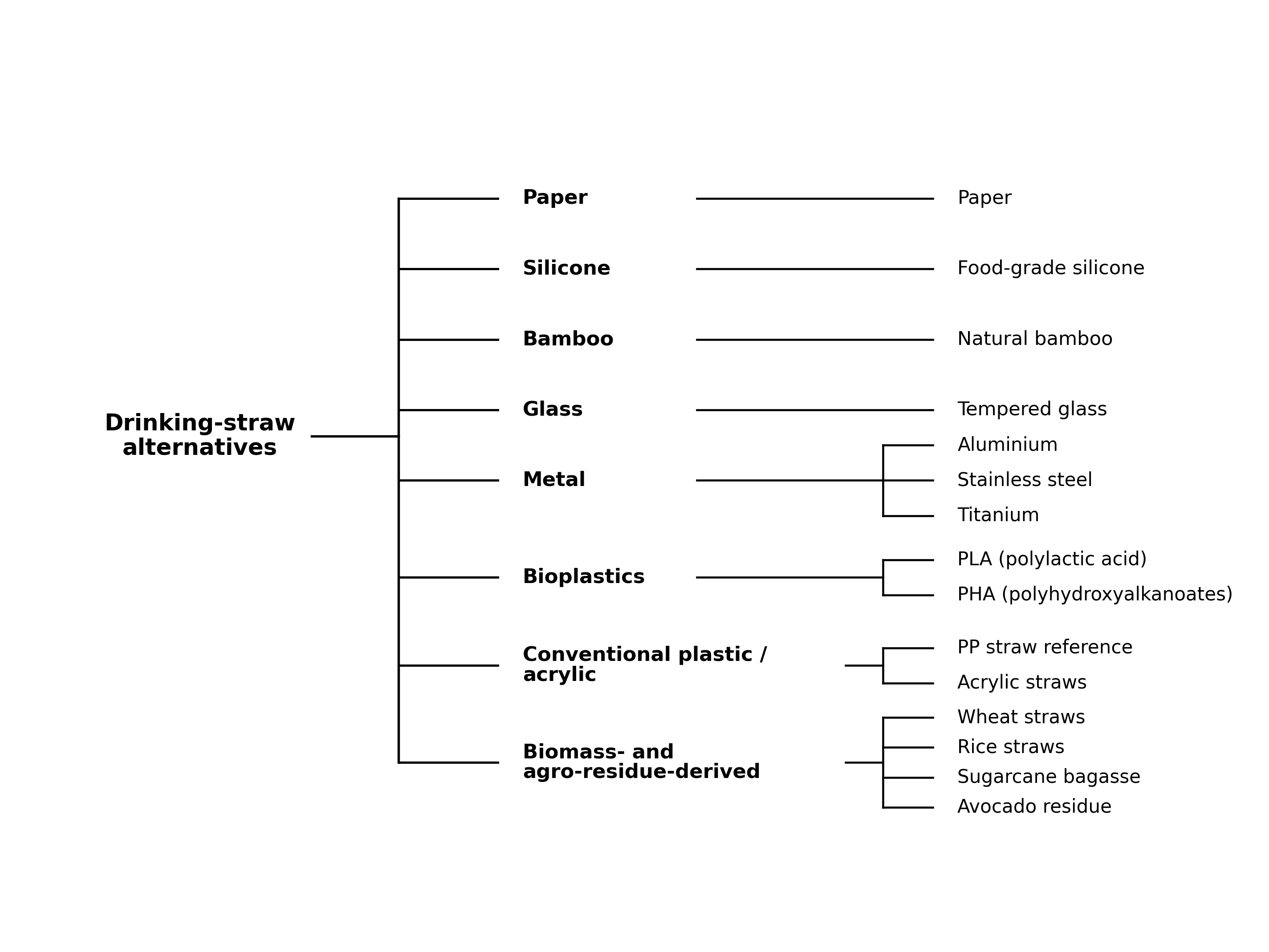
**Supplementary Fig. S3. Taxonomy of drinking-straw alternatives considered in this study,** grouped into paper, silicone, bamboo, glass, metal, bioplastics, conventional plastic/acrylic, and biomass- or agro-residue-derived materials.


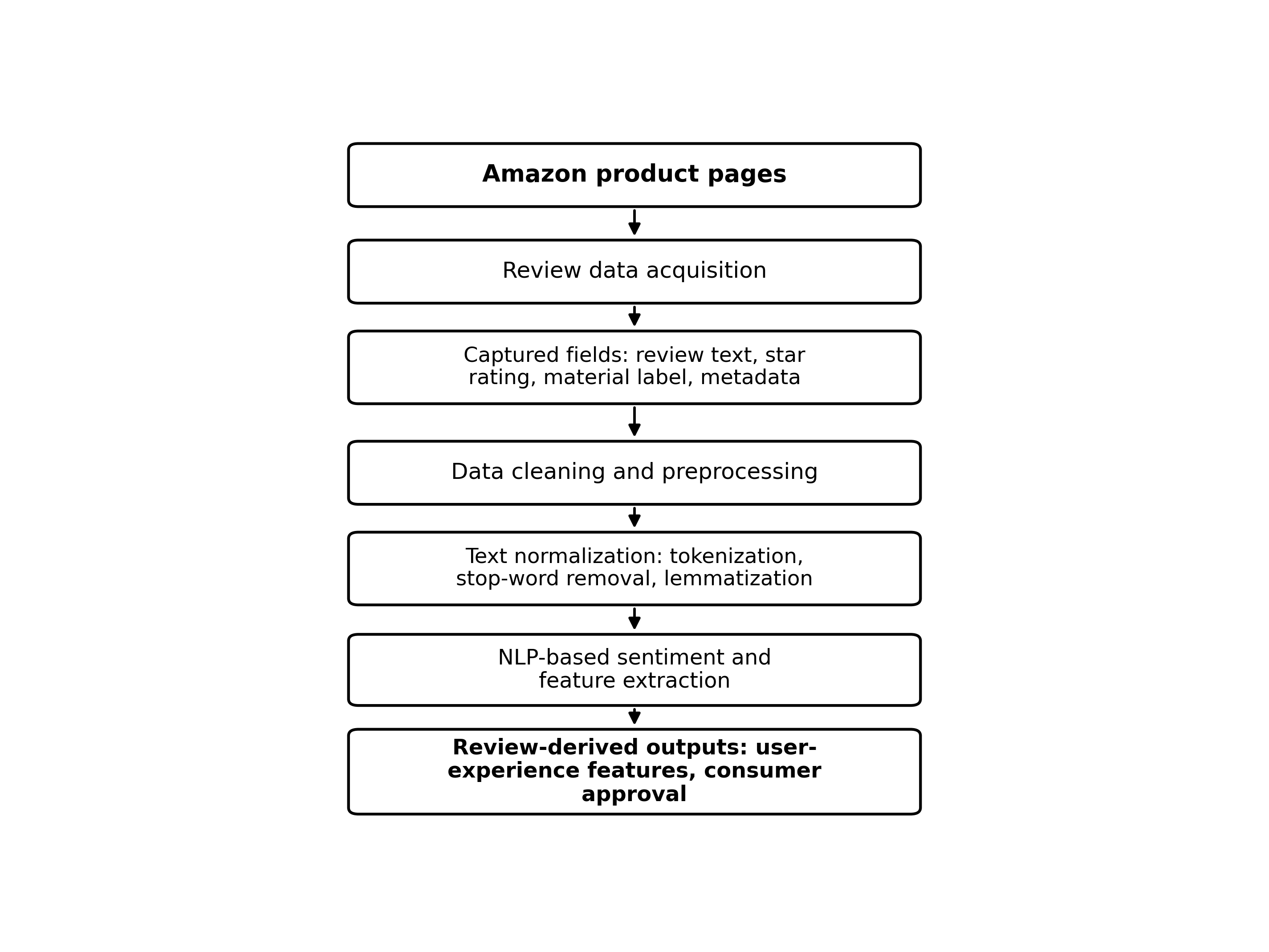


**Supplementary Fig. S4.** Conceptual workflow for acquiring and preprocessing review-derived user evidence from Amazon product pages. The workflow summarizes product-page identification, review data acquisition, captured metadata, data cleaning, text normalization, NLP-based sentiment and feature extraction, and generation of review-derived user-experience features and consumer-approval outputs.

| (A) | (B) |
| --- | --- |
| 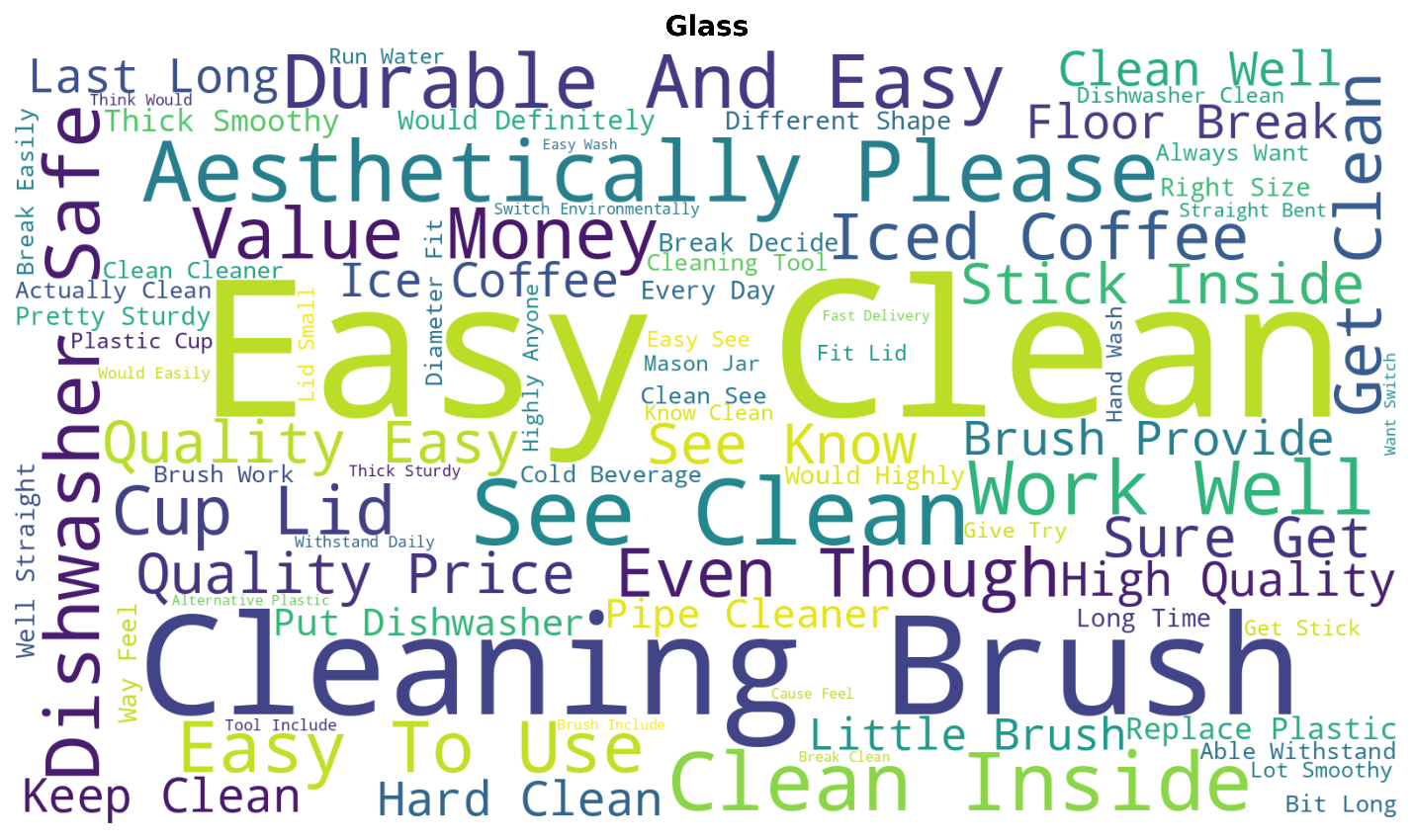 | 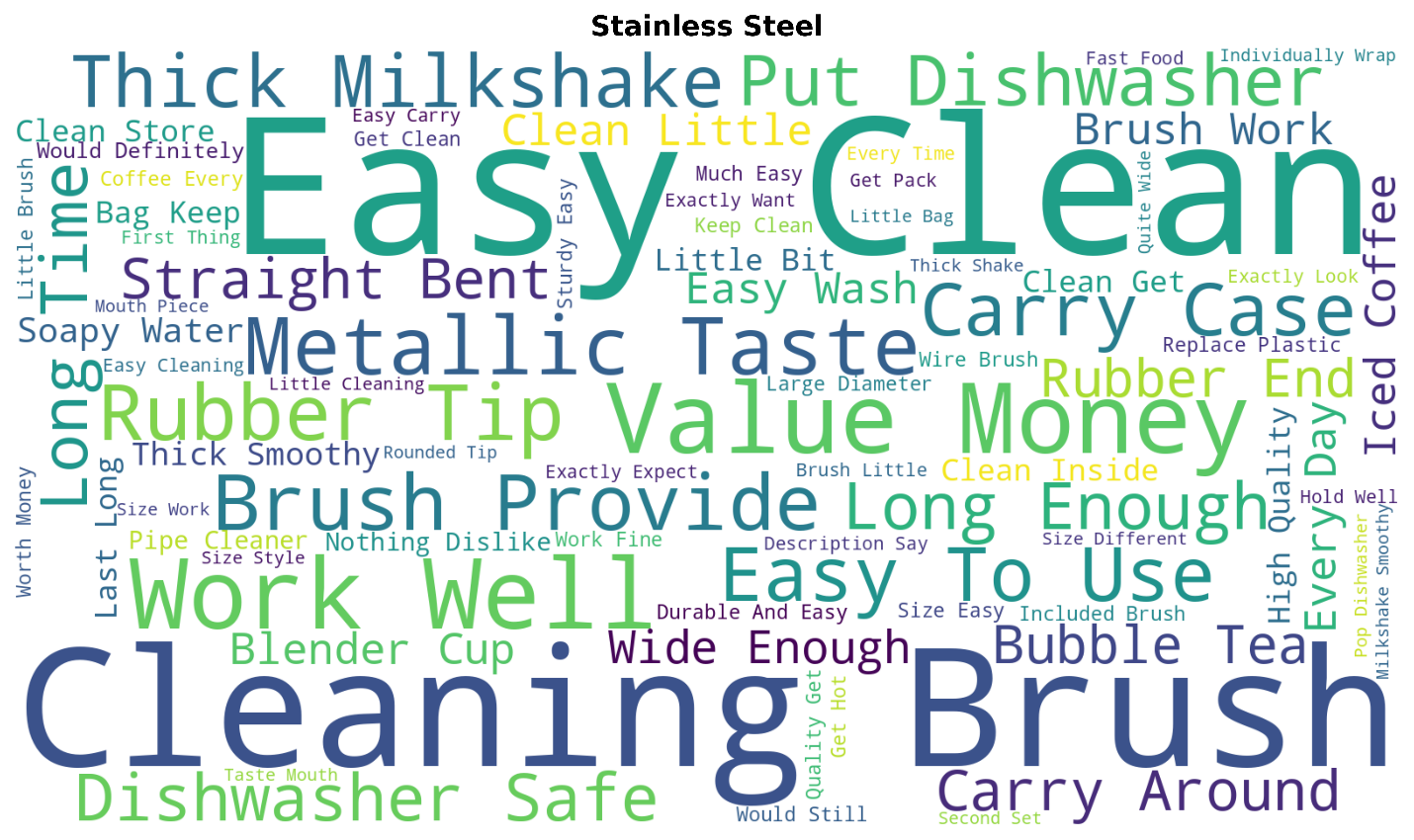 |
| **(C)** | **(D)** |
| 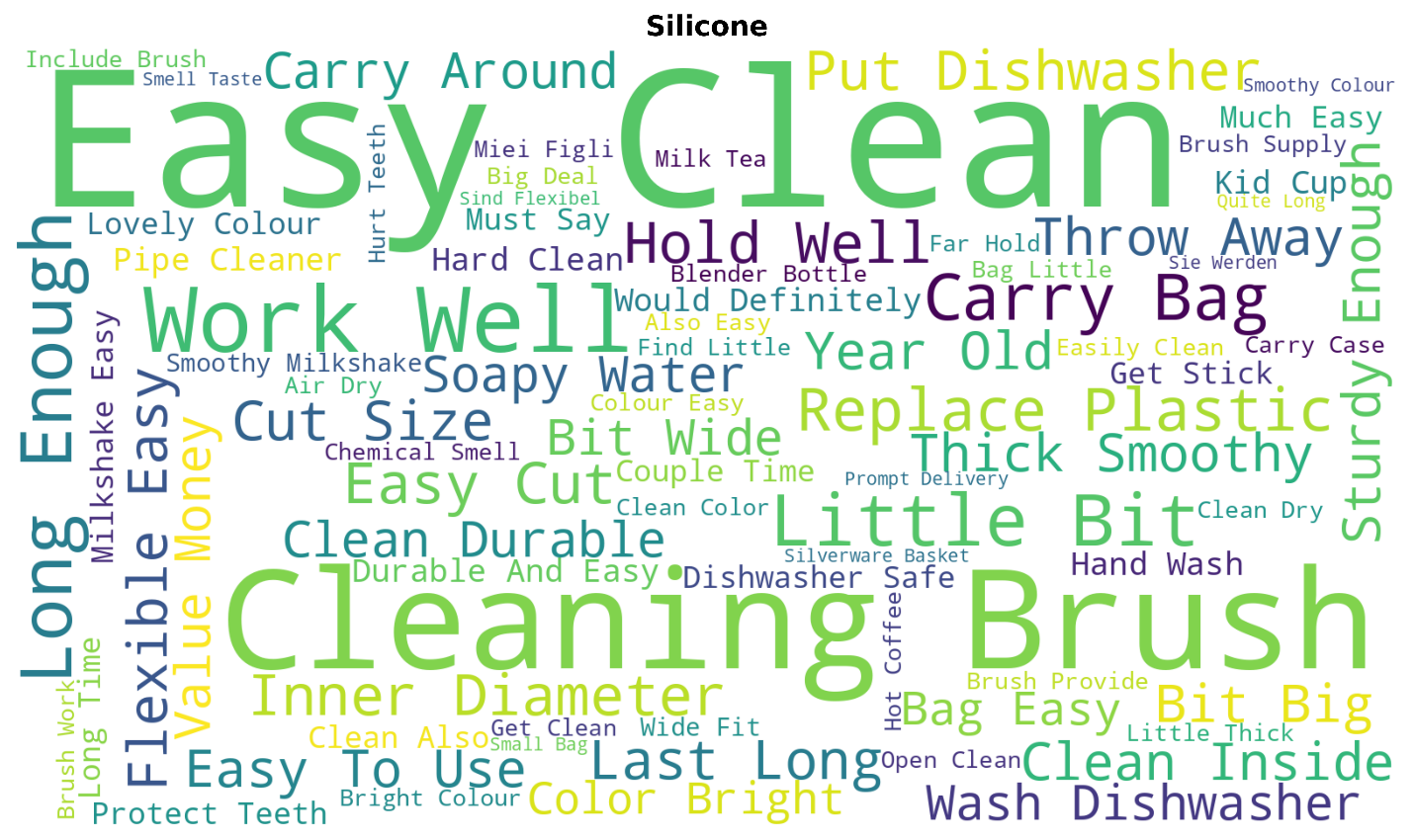 | 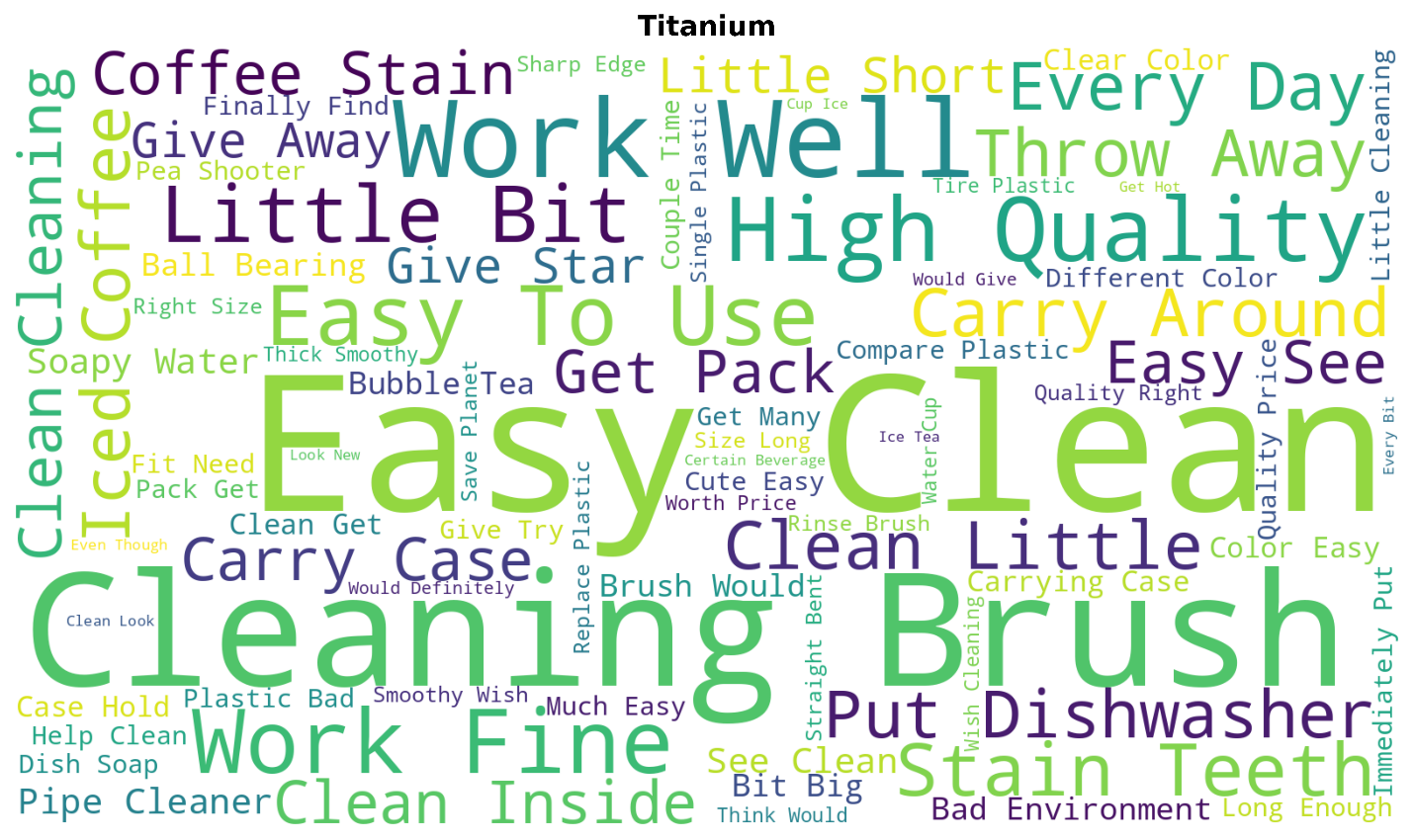 |
| **(E)** |  |
| 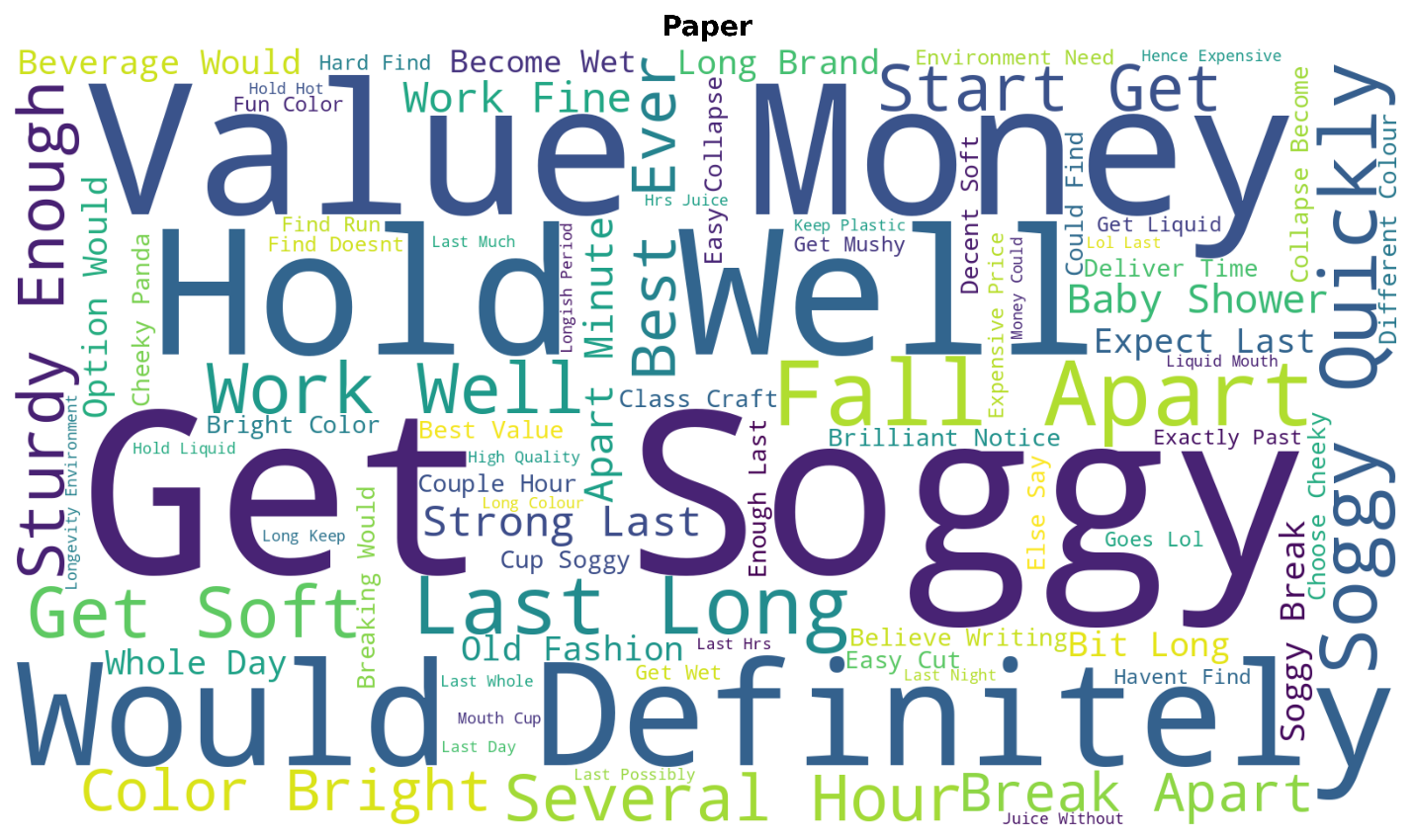 | |

**Supplementary Fig. S5. Word clouds summarizing frequent informative terms in online customer reviews collected from Amazon product pages for the five shortlisted straw materials: (**A) Glass, (B) Stainless Steel, (C) Silicone, (D) Titanium, and (E) Paper. Larger words indicate higher term frequency after text preprocessing and removal of non-informative terms.

**Supplementary S3. Integrated dataset, attribute encoding, and MCDA weighting schemes**

This Supplementary section provides the extended procedural details supporting the condensed Methods and Results sections in the main manuscript, including review acquisition, pre-processing, feature extraction, GHG harmonization assumptions, and scenario-specific scoring and explainability settings.

## Supplementary Note S3. Data collection and pre-processing (reviews/NLP)

**Sources and scope**: Amazon.co.uk product pages for reusable and biodegradable straws across 14 material categories matching Table S1; peer-reviewed LCA studies and sustainability reports (2014-2025).

**Acquisition:** Review acquisition followed a two-stage Python workflow. First, product names and product-page links were collected from Amazon.co.uk search and product pages using material-specific search terms. Second, a review-extraction script iterated through the collected product-page links and extracted available review text, star rating, timestamp, material label, metadata, and helpful-vote information where available.

**Cleaning:** remove duplicates, punctuation, and stopwords; exclude malformed entries; tokenize and lemmatize. Neutral reviews were excluded where appropriate to improve contrast between clearly positive and clearly negative user experiences during polarity-based comparisons, while rating-based consumer approval indicators were retained separately in the integrated assessment.

**Sentiment & features:** lexicon-based tools (TextBlob, VADER) for polarity; aspect signals for bendability, heat/cold tolerance, absence of adverse smell/taste effects, child safety, and travel/driver safety; additional descriptive features include word-frequency and material-specific review vocabulary.

Quality control: Listings were de-duplicated across sellers/variants; outliers and off-topic reviews were removed by manual inspection to avoid biasing consumer approval shares.

**Integration and modelling:** literature-derived per-use GHG indicators were normalized to a per-use basis and then integrated with review-derived user features through a scenario-weighted MCDA layer. Decision-tree rules were used for explainability, and association rules were mined as descriptive complements.

| **Table S3a.** Descriptive screening of straw-material categories and selected user-experience attributes used to support material selection, including conventional plastic as a reference baseline. | | | | | | | | | |
| --- | --- | --- | --- | --- | --- | --- | --- | --- | --- |
| **Straw category** | | **Material** | **Bendability** | **Effects of heat and cold** | **No smell/taste effect** | **Child safety** | | **Travel/driver safety** | **Favourable attribute count** |
| Conventional plastic | | | ***✔*** | ***No*** | ✔ | ***✔*** | ***✔*** | | ***5*** |
| Paper-based | | | No | ✔ | No | ✔ | ✔ | | 2 |
| Bamboo-based | Bamboo (Reusable) | | No | No | ✔ | No | No | | 2 |
| Bamboo-based | Bamboo (Disposable) | | No | ✔ | No | No | No | | 0 |
| Glass | | | No | No | ✔ | No | No | | 2 |
| Acrylic (hard plastic) | | | No | No | ✔ | ✔ | ✔ | | 4 |
| Silicone | | | ✔ | No | No | ✔ | ✔ | | 4 |
| Metal-based | Aluminium | | No | ✔ | ✔ | No | No | | 1 |
|  | Stainless steel | | No | ✔ | ✔ | No | No | | 1 |
|  | Titanium | | No | No | ✔ | No | No | | 2 |
| Bioplastics | PLA (Polylactic Acid) | | ✔ | No | ✔ | ✔ | ✔ | | 5 |
| Biomass/agro-residue-derived | Avocado | | ✔ | No | ✔ | ✔ | ✔ | | 5 |
| Biomass/agro-residue-derived | Wheat straws | | No | ✔ | No | ✔ | ✔ | | 2 |
| Biomass/agro-residue-derived | Rice straws | | No | ✔ | No | ✔ | ✔ | | 2 |
| Biomass/agro-residue-derived | Sugarcane | | No | No | ✔ | No | No | | 2 |
| ***Note:*** *A check mark (✓) indicates the presence of a favourable attribute, whereas “No” indicates its absence or an unfavourable outcome. The favourable attribute count is a descriptive screening measure and is not equivalent to the integrated MCDA score. Conventional plastic is included as a reference baseline only and was not included in the integrated MCDA ranking because comparable review-derived user-experience and consumer approval evidence was not consistently available across the same evidence domains used for the shortlisted alternatives.* | | | | | | | | | |

| Table S3b. Policy-scenario weight settings and integrated MCDA score equations | | | | |
| --- | --- | --- | --- | --- |
| Scenario | **Weight** | | | **Integrated MCDA score equation** |
|  | **GHG-derived score weight** | **User-experience feature score weight** | **Consumer-approval score weight** |  |
| Balanced (1/3 each) | 0.333 | 0.333 | 0.333 | 0.333×GHG-derived score+0.333×User-experience feature score+0.333×consumer approval |
| GHG-prioritized (0.5,0.3,0.2) | 0.500 | 0.300 | 0.200 | 0.500×GHG-derived score+0.300×User-experience feature score+0.200×consumer approval |
| User-experience-prioritized (0.3,0.5,0.2) | 0.300 | 0.500 | 0.200 | 0.300×GHG-derived score+0.500×User-experience feature score+0.200×consumer approval |
| No-approval (0.6,0.4,0.0) | 0.600 | 0.400 | 0.000 | 0.600×GHG-derived score + 0.400×User-experience feature score |

## Supplementary S4. MCDA scoring, ranking, decision-tree-based explainability, and association-rule settings

**Integrated MCDA score:** A higher integrated MCDA score indicates stronger combined performance within the evaluated criteria and scenario-specific weights across the three evidence components used in this study: the GHG-derived score, the user-experience feature score, and consumer approval. It does not indicate the overall sustainability of a material. The weighted-sum MCDA structure follows standard multi-criteria decision-analysis practice [50,51].

The weighting scenarios were predefined decision-priority scenarios rather than data-fitted parameters or universal sustainability weights. They were used to examine the stability of material rankings under alternative plausible decision contexts. The balanced scenario provides a neutral equal-weight benchmark. The GHG-prioritized scenario represents a decision context emphasizing per-use climate-related performance. The user-experience-prioritized scenario represents a context emphasizing usability and functional performance. The no-approval scenario removes consumer approval as a sensitivity case to test whether the rankings remain supported by the GHG-derived score and user-experience feature score. These scenarios are therefore used for transparent sensitivity-oriented interpretation rather than proposed as a new MCDA method.

**GHG-derived score:** The GHG-derived score was calculated by min–max inversion of the literature-derived per-use GHG indicator, so that lower per-use GHG values corresponded to higher GHG-derived scores.

**User-experience feature score:** The user-experience feature score was calculated as the mean of five binary favourable usability and safety attributes: bendability, heat/cold tolerance, absence of adverse smell/taste effects, child safety, and travel/driver safety. A value of 1 indicated the presence of a favourable attribute and 0 indicated its absence. This simplified coding did not represent the intensity, frequency, severity, or context of reported user-experience concerns.

**Consumer approval:** Consumer approval represents the share of positive reviews, defined as ratings ≥4/5 stars, averaged per material where review-count weighting was applicable.

**Decision-tree-based explainability:** A shallow DecisionTreeRegressor was fitted as a post hoc explanatory tool using the per-use GHG indicator and user-experience feature score as explanatory variables and the GHG-prioritized integrated MCDA score as the target. The model was configured with a maximum depth of two and random_state = 42. Consumer approval contributed to the integrated target score through its scenario weight of 0.20 but was not supplied as a separate explanatory variable. The fitted split thresholds were 0.0805 kg CO₂e/use and 0.600; these were displayed in Fig. 4 as 0.081 kg CO₂e/use and 0.600, respectively. The tree was used to summarize the calculated MCDA results and was not treated as an independently validated predictive model or as evidence of externally generalizable thresholds [52].

**Association-rule settings and results:** Apriori association-rule mining was applied only to the five binary user-experience and safety attributes: bendability, heat/cold tolerance, absence of adverse smell/taste effects, child safety, and driving/travel safety [53]. The minimum support and confidence thresholds were 0.4 and 0.7, respectively. Support represents the proportion of evaluated materials containing an item combination, confidence represents the conditional occurrence of the consequent given the antecedent, and lift compares the observed co-occurrence with that expected under independence. Because the analysis included five materials, a support value of 0.4 corresponded to co-occurrence in two materials. Two directional rules met the specified thresholds: child safety → driving/travel safety and driving/travel safety → child safety. Each rule had support = 0.40, confidence = 1.00, and lift = 2.50. Given the five-material matrix and simplified binary coding, these rules were interpreted only as exploratory descriptive co-occurrence patterns. They were not used to support material rankings, consumer-approval conclusions, prediction, causal inference, external generalization, or modification of the MCDA scores. The retained rules are summarized in Table S4.5.

| Table S4.1. MCDA results — Balanced (1/3 each): GHG-derived score, User-experience feature score, consumer approval, Integrated MCDA score, Rank for each material | | | | | |
| --- | --- | --- | --- | --- | --- |
| **Material** | **GHG-derived score** | **User-experience feature score** | **consumer approval** | **Integrated MCDA score** | **Rank** |
| Silicone | 0.980 | 0.800 | 0.790 | 0.857 | 1 |
| Glass | 0.956 | 0.400 | 0.815 | 0.724 | 2 |
| Titanium | 1.000 | 0.400 | 0.680 | 0.693 | 3 |
| Stainless steel | 0.883 | 0.200 | 0.800 | 0.628 | 4 |
| Paper | 0.000 | 0.400 | 0.685 | 0.362 | 5 |

## Table S4.2. MCDA results — GHG-prioritized (0.5, 0.3, 0.2)

| **Material** | **GHG-derived score** | **User-experience feature score** | **consumer approval** | **Integrated MCDA score** | **Rank** |
| --- | --- | --- | --- | --- | --- |
| Silicone | 0.980 | 0.800 | 0.790 | 0.888 | 1 |
| Glass | 0.956 | 0.400 | 0.815 | 0.761 | 2 |
| Titanium | 1.000 | 0.400 | 0.680 | 0.756 | 3 |
| Stainless steel | 0.883 | 0.200 | 0.800 | 0.662 | 4 |
| Paper | 0.000 | 0.400 | 0.685 | 0.257 | 5 |

**Table S4.3.** MCDA results — User-experience-prioritized (0.3, 0.5, 0.2)

| **Material** | **GHG-derived score** | | **User-experience feature score** | **consumer approval** | **Integrated MCDA score** | **Rank** |
| --- | --- | --- | --- | --- | --- | --- |
| Silicone | | 0.980 | 0.800 | 0.790 | 0.852 | 1 |
| Glass | | 0.956 | 0.400 | 0.815 | 0.650 | 2 |
| Titanium | | 1.000 | 0.400 | 0.680 | 0.636 | 3 |
| Stainless steel | | 0.883 | 0.200 | 0.800 | 0.525 | 4 |
| Paper | | 0.000 | 0.400 | 0.685 | 0.337 | 5 |

**Table S4.4**. MCDA results — No-approval (0.6, 0.4, 0.0)

| **Material** | GHG-derived score | User-experience feature score | consumer approval | Integrated MCDA score | **Rank** |
| --- | --- | --- | --- | --- | --- |
| Silicone | 0.980 | 0.800 | 0.790 | 0.908 | 1 |
| Titanium | 1.000 | 0.400 | 0.680 | 0.760 | 2 |
| Glass | 0.956 | 0.400 | 0.815 | 0.734 | 3 |
| Stainless steel | 0.883 | 0.200 | 0.800 | 0.610 | 4 |
| Paper | 0.000 | 0.400 | 0.685 | 0.160 | 5 |

**Decision tree — GHG-prioritized scenario (0.5, 0.3, 0.2)**

The GHG-prioritized MCDA scenario was calculated as:

Integrated MCDA score = 0.500 × GHG-derived score + 0.300 × User-experience feature score + 0.200 × consumer approval

The shallow decision-tree analysis produced the following interpretable rules:

|--- Per-use GHG indicator ≤ 0.081 kg CO₂e/use
| |--- User-experience feature score > 0.600
| | |--- Highest integrated MCDA score / Silicone case
| |--- User-experience feature score ≤ 0.600
| | |--- Intermediate integrated MCDA scores / Glass, Titanium, and Stainless steel cases
|--- Per-use GHG indicator > 0.081 kg CO₂e/use
| |--- Lowest integrated MCDA score / Paper case

The primary split at a per-use GHG indicator of 0.081 kg CO₂e/use separated Paper from the four lower-per-use-GHG reusable alternatives within the evaluated five-material decision matrix. This split is an internal explanatory threshold and not a universal environmental benchmark.

Within the lower-per-use-GHG group, the user-experience feature-score threshold of 0.600 separated Silicone from Glass, Titanium, and Stainless steel. This threshold is specific to the evaluated inputs and GHG-prioritized scenario and should not be interpreted as a general usability benchmark.

The decision tree used the aggregated user-experience feature score rather than the five binary attributes individually. It was retained solely as a concise explanation of the calculated GHG-prioritized MCDA pattern and not as a predictive model, an independent validation of the MCDA, or a basis for external generalization.

**Table S4.5.** Association rules meeting the predefined support and confidence thresholds.

| **Antecedent** | **Consequent** | **Support** | **Confidence** | **Lift** |
| --- | --- | --- | --- | --- |
| Child safety | Driving/travel safety | 0.40 | 1.00 | 2.50 |
| Driving/travel safety | Child safety | 0.40 | 1.00 | 2.50 |

**Supplementary Table S5**

| **Supplementary Table S5.** Top five cleaned informative bigrams and frequencies extracted from positive-review and negative-review subsets for each shortlisted straw material. | | | | |
| --- | --- | --- | --- | --- |
| **Material** | **Positive Bigram** | **Positive Frequency** | **Negative Bigram** | **Negative Frequency** |
| Glass | Easy to Clean | 112 | Feels Weird | 4 |
|  | Cleaning Brush | 45 | Less Cold | 4 |
|  | Aesthetically Pleasing | 14 | Mouthpiece Thickness | 4 |
|  | Clean Inside | 13 | Thick Feel | 4 |
|  | Dishwasher Safe | 11 | Wish Thicker | 4 |
|  | **Positive Top-5 Total** | **195** | **Negative Top-5 Total** | **20** |
| Stainless steel | Easy to Clean | 179 | Cleaning Brush | 4 |
|  | Cleaning Brush | 117 | Normal Size | 4 |
|  | Value for Money | 26 | Carry Around | 3 |
|  | Works Well | 26 | Long Brush | 3 |
|  | Metallic Taste | 17 | Too Long | 3 |
|  | **Positive Top-5 Total** | **365** | **Negative Top-5 Total** | **17** |
| Silicone | Easy to Clean | 227 | Smell/Taste | 6 |
|  | Cleaning Brush | 88 | Plasticky Feel | 5 |
|  | Works Well | 33 | Plasticky Smell | 5 |
|  | Long Enough | 19 | Ice Cold | 4 |
|  | Carry Bag | 15 | Bit Wide | 3 |
|  | **Positive Top-5 Total** | **382** | **Negative Top-5 Total** | **23** |
| Titanium | Easy to Clean | 58 | Carry Around | 2 |
|  | Cleaning Brush | 34 | Little Short | 2 |
|  | Works Well | 14 | Around Keychain | 1 |
|  | High Quality | 9 | Break Time | 1 |
|  | Super Easy | 8 | Brush Problem | 1 |
|  | **Positive Top-5 Total** | **123** | **Negative Top-5 Total** | **7** |
| Paper | Gets Soggy | 33 | Couple Hours | 5 |
|  | Value for Money | 23 | Cup Sogginess | 5 |
|  | Holds Well | 21 | Gets Soggy Quickly | 5 |
|  | Falls Apart | 19 | Quickly Last | 5 |
|  | Lasts Long | 12 | Bad Papery | 4 |
|  | **Positive Top-5 Total** | **108** | **Negative Top-5 Total** | **24** |

*Note:* *Frequencies correspond to the cleaned bigram-frequency plots shown in Fig. 1 and Fig. 2. The table reports the top-five cleaned informative bigrams retained after applying the same filtering procedure used for the lexical figures, including removal of material names, generic product terms, brand/product names, and repetitive non-informative expressions. Where multiple bigrams had the same frequency, the retained entries reflect the cleaned frequency output after excluding non-informative or brand-related terms. The positive-review and negative-review subsets refer to review-level sentiment classes, not necessarily to the standalone polarity of each bigram. A negative-sounding expression may therefore occur in the positive-review subset when it appears within a review whose overall classification is positive, including mixed evaluations or references to an absent or reduced problem. The “Top-5 total” values represent the sum of the five listed bigram frequencies only and should not be interpreted as total review counts or total sentiment counts.*

## Supplementary References

**The following sources are cited in the Supplementary Information or provide the material-specific source entries for the review-derived dataset.**

[25] Amazon.co.uk (n.d.-a). Paper drinking straws on Amazon.co.uk. https://www.amazon.co.uk/s?k=paper+drinking+straws. Accessed 28 September 2025.

[26] Amazon.co.uk (n.d.-b). Bamboo straws on Amazon.co.uk. https://www.amazon.co.uk/s?k=bamboo+straws. Accessed 28 September 2025.

[27] Amazon.co.uk (n.d.-c). Glass drinking straws on Amazon.co.uk. https://www.amazon.co.uk/s?k=glass+drinking+straws. Accessed 28 September 2025.

[28] Amazon.co.uk (n.d.-d). Acrylic drinking straws on Amazon.co.uk. https://www.amazon.co.uk/s?k=acrylic+drinking+straws. Accessed 28 September 2025.

[29] Amazon.co.uk (n.d.-e). Silicone straws on Amazon.co.uk. https://www.amazon.co.uk/silicone-straws/s?k=silicone+straws. Accessed 28 September 2025.

[30] Amazon.co.uk (n.d.-f). Aluminium drinking straws on Amazon.co.uk. https://www.amazon.co.uk/s?k=aluminium+drinking+straws. Accessed 28 September 2025.

[31] Amazon.co.uk (n.d.-g). Stainless steel drinking straws on Amazon.co.uk. https://www.amazon.co.uk/s?k=stainless+steel+straws. Accessed 28 September 2025.

[32] Amazon.co.uk (n.d.-h). Titanium drinking straws on Amazon.co.uk. https://www.amazon.co.uk/s?k=titanium+drinking+straws. Accessed 28 September 2025.

[33] Amazon.co.uk (n.d.-i). PLA drinking straws on Amazon.co.uk. https://www.amazon.co.uk/s?k=pla+drinking+straws. Accessed 28 September 2025.

[34] Amazon.co.uk (n.d.-j). PHA drinking straws on Amazon.co.uk. https://www.amazon.co.uk/s?k=pha+drinking+straws. Accessed 28 September 2025.

[35] Amazon.co.uk (n.d.-k). Avocado drinking straws on Amazon.co.uk. https://www.amazon.co.uk/s?k=avocado+drinking+straws. Accessed 28 September 2025.

[36] Amazon.co.uk (n.d.-l). Wheat drinking straws on Amazon.co.uk. https://www.amazon.co.uk/s?k=wheat+drinking+straws. Accessed 28 September 2025.

[37] Amazon.co.uk (n.d.-m). Rice drinking straws on Amazon.co.uk. https://www.amazon.co.uk/s?k=rice+drinking+straws. Accessed 28 September 2025.

[38] Amazon.co.uk (n.d.-n). Sugarcane drinking straws on Amazon.co.uk. https://www.amazon.co.uk/s?k=sugar+cane+drinking+straws. Accessed 28 September 2025.

[39] Boonniteewanich, J., Pitivut, S., Tongjoy, S., Lapnonkawow, S. and Suttiruengwong, S., 2014. Evaluation of carbon footprint of bioplastic straw compared to petroleum based straw products. *Energy Procedia*, *56*, pp.518-524. <https://doi.org/10.1016/j.egypro.2014.07.184>.

[40] Zanghelini, G.M., Cherubini, E., Dias, R., Kabe, Y.H.O. and Delgado, J.J.S., 2020. Comparative life cycle assessment of drinking straws in Brazil. *Journal of cleaner production*, *276*, p.123070. <https://doi.org/10.1016/j.jclepro.2020.123070>.

[41] Chitaka, T.Y., Russo, V. and von Blottnitz, H., 2020. In pursuit of environmentally friendly straws: a comparative life cycle assessment of five straw material options in South Africa. *The International Journal of Life Cycle Assessment*, *25*(9), pp.1818-1832. <https://doi.org/10.1007/s11367-020-01786-w>.

[42] Moy, C.H., Tan, L.S., Shoparwe, N.F., Shariff, A.M. and Tan, J., 2021. Comparative study of a life cycle assessment for bio-plastic straws and paper straws: Malaysia’s perspective. *Processes*, *9*(6), p.1007. <https://doi.org/10.3390/pr9061007>.

[43] Gao, A.L. and Wan, Y., 2022. Life cycle assessment of environmental impact of disposable drinking straws: A trade-off analysis with marine litter in the United States. *Science of the Total Environment*, *817*, p.153016. <https://doi.org/10.1016/j.scitotenv.2022.153016>.

[44] Guo, X., Li, C., Wang, Y. and Liu, X., 2023. Multidimensional evaluation for environment impacts of disposable drinking straws. Journal of Cleaner Production, 387, 135756. <https://doi.org/10.1016/j.jclepro.2023.135756>.

[45] Xie, Y., Ji, Z., Abdalkarim, S.Y.H., Huang, H., Yunusov, K.E. and Yu, H.Y., 2024. Investigating interface adhesion of PLA-coated cellulose paper straws: degradation, plant growth effects, and life cycle assessment. Journal of Hazardous Materials, 480, 136101. https://doi.org/10.1016/j.jhazmat.2024.136101.

[46] Rai, R., Ranjan, R., Kant, C. and Dhar, P., 2024. Microplastic and adhesive free, multifunctional, circular economy approach-based biomass-derived drinking straws. *Iscience*, *27*(5). <https://doi.org/10.1016/j.isci.2024.109630>.

[47] Eleni, P. and Boukouvalas, C., 2025. Environmental and Economic Impacts of Substituting Single-Use Plastic Straws: A Life-Cycle Assessment for Greece. *Polymers*, *17*(9), p.1235. <https://doi.org/10.3390/polym17091235>.

[48] U.S. Department of Energy (2017). Bandwidth study on energy use and potential energy saving opportunities in U.S. titanium manufacturing. U.S. Department of Energy, Washington, DC.

[49] IperionX Limited (2023). IperionX releases life cycle assessment of 100% recycled titanium powder: confirms potential for market-leading low-carbon titanium powder production. ASX announcement, 26 April 2023. Accessed 5 October 2025.

[50] Keeney, R.L. and Raiffa, H., 1993. *Decisions with multiple objectives: preferences and value trade-offs*. Cambridge university press. <https://doi.org/10.1017/CBO9781139174084>.

[51] Triantaphyllou, E., 2000. Multi-Criteria Decision Making Methods: A Comparative Study. Springer, Boston, MA. <https://doi.org/10.1007/978-1-4757-3157-6>.

[52] Pedregosa, F., Varoquaux, G., Gramfort, A., Michel, V., Thirion, B., Grisel, O., Blondel, M., Prettenhofer, P., Weiss, R., Dubourg, V. and Vanderplas, J., 2011. Scikit-learn: Machine learning in Python. Journal of Machine Learning Research, 12, pp. 2825–2830. <https://doi.org/10.5555/1953048.2078195>.

[53] Agrawal, R., Imieliński, T. and Swami, A., 1993. Mining association rules between sets of items in large databases. In Proceedings of the 1993 ACM SIGMOD International Conference on Management of Data, pp. 207–216. <https://doi.org/10.1145/170035.170072>.
